# Evolution of *NLR* Resistance Genes in Magnoliids: Dramatic Expansions of *CNLs* and Multiple Losses of *TNLs*

**DOI:** 10.3389/fpls.2021.777157

**Published:** 2021-12-21

**Authors:** Jia-Yi Wu, Jia-Yu Xue, Yves Van de Peer

**Affiliations:** ^1^College of Horticulture, Academy for Advanced Interdisciplinary Studies, Nanjing Agricultural University, Nanjing, China; ^2^State Key Laboratory of Palaeobiology and Stratigraphy, Nanjing Institute of Geology and Palaeontology (CAS), Nanjing, China; ^3^Department of Plant Biotechnology and Bioinformatics, VIB-UGent Center for Plant Systems Biology, Ghent University, Ghent, Belgium; ^4^Department of Biochemistry, Genetics and Microbiology, University of Pretoria, Pretoria, South Africa

**Keywords:** magnoliids, *NLR* genes, phylogeny, evolution pattern, expression

## Abstract

Magnoliids are the third-largest group of angiosperms and occupy a critical position in angiosperm evolution. In the past years, due to the lack of sequenced genomes, the disease resistance gene (*R* gene) profile of magnoliids remains poorly understood. By the genome-wide identification of 1,832 *NLR* genes from seven magnoliid genomes, we built a framework for the evolution of magnoliid *R* genes. *TNL* genes were completely absent from five magnoliids, presumably due to immune pathway deficiencies. A total of 74 ancestral *R* genes (70 *CNLs*, 3 *TNLs*, and 1 *RNL*) were recovered in a common ancestor of magnoliids, from which all current *NLR* gene repertoires were derived. Tandem duplication served as the major drive for *NLR* genes expansion in seven magnoliid genomes, as most surveyed angiosperms. Due to recent rapid expansions, most magnoliids exhibited “a first expansion followed by a slight contraction and a further stronger expansion” evolutionary pattern, while both *Litsea cubeba* and *Persea americana* showed a two-times-repeated pattern of “expansion followed by contraction.” The transcriptome analysis of seven different tissues of *Saururus chinensis* revealed a low expression of most *NLR* genes, with some *R* genes displaying a relatively higher expression in roots and fruits. Overall, our study sheds light on the evolution of *NLR* genes in magnoliids, compensates for insufficiency in major angiosperm lineages, and provides an important reference for a better understanding of angiosperm *NLR* genes.

## Introduction

Plants have faced numerous destructive pathogens throughout their evolutionary history, such as bacteria, viruses, and fungi. Over time, plants have adapted and employed a two-tiered versatile immune system to ward off the perturbations of pathogens. The first tier, described as the pathogen-associated molecular pattern (PAMP)-triggered immunity (PTI), recognizes PAMPs with cell membrane-localized receptors. The second tier, known as effector-triggered immunity (ETI), perceives pathogen-derived effectors through diverse strategies *via* intracellular disease resistance genes (*R* genes), ultimately resulting in resistance responses typically accompanied by hypersensitive reactions (HRs) in infected parts of the plants (Cui et al., 2015; Jones et al., 2016; Wang and Chai, 2020).

Nucleotide-binding site-leucine-rich repeat (*NLR* or *NBS-LRR*) genes comprise the largest group of plant *R* genes (Kourelis and van der Hoorn, 2018). The encoded NLR proteins are usually found in a signaling-competent, autoinhibited state with the LRR domain folding back onto the central NBS domain (Hu et al., 2013). NLR proteins are activated on the recognition of invading pathogens, and the NBS domain undergoes conformational alterations, as it possesses exposed N-terminal domains that trigger downstream HRs that elicit the apoptosis of infected cells to prevent pathogen transmission and proliferation (Andersen et al., 2018).

Angiosperm *NLR* genes can be divided into three subclasses, namely, *TIR-NBS-LRR* (*TNL*), *CC-NBS-LRR* (*CNL*), and *RPW8-NBS-LRR* (*RNL*), based on the identity of the N-terminal domain, which can be one of the three types, namely, Toll/Interleukin-1 receptor-like (TIR), coiled-coil (CC), and resistance to powdery mildew 8 (RPW8; Parker et al., 1997; Pan et al., 2000; Shao et al., 2014). Most TNL and CNL proteins function as pathogen sensors, either directly recognizing pathogenic effector proteins or indirectly monitoring the status shift of host proteins targeted by effectors (Kourelis and van der Hoorn, 2018). RNL proteins assist the downstream immune signal transduction of TNL and CNL proteins and are thus termed as the “helper” NLR (Jubic et al., 2019; Wang and Chai, 2020). Additionally, with regard to the molecular mechanism of NLR proteins that regulate cell death and resistance downstream, it has been well documented that CNL and RNL proteins act as Ca^2+^-permeable channels that trigger immunity and cell necrosis (Bi et al., 2021; Jacob et al., 2021).

The *NLR* genes originated from the common ancestor of all green plants and have been found in green algae and bryophytes (Xue et al., 2012; Yue et al., 2012; Shao et al., 2019). The divergence of *NLR* genes occurred early on, and the *CNL* and *TNL* subclasses have also been found in green algae and bryophytes, while other plant taxa have specific subclasses of their own [e.g., a/b-hydrolase-NBS-LRR (*HNL*) in liverworts and protein-kinase-NBS-LRR (*PNL*) in mosses] (Xue et al., 2012). Studies on lycophyte and fern *NLRs* are rare, and no new subclasses have been found in these taxa. Gymnosperms possess three similar *NLR* subclasses as angiosperms, namely, *CNL*, *TNL*, and *RNL* (Shao et al., 2019). The evolutionary history of *NLR* genes in angiosperms proceeded in two stages. The first was a salient stage starting at the origin of angiosperms until the Cretaceous-Paleogene (K-Pg) boundary when *NLR* genes were kept in low gene numbers. The second was a drastic expanding stage after the K-Pg boundary that led to the large *NLR* gene numbers we observed at present (Shao et al., 2016).

Due to frequent gene duplication and loss events, different angiosperm taxa possess different *NLR* gene numbers [e.g., rice has 498 *NLRs* (497 *CNLs* and 1 *RNL* but no *TNLs*); *Arabidopsis thaliana* has 165 *NLRs* (52 *CNLs*, 106 *TNLs*, and 7 *RNLs*), and tomato has 255 *NLRs* (222 *CNLs*, 31 *TNLs*, and 2 *RNLs*)] (Shao et al., 2016; Liu et al., 2021). The absence of *TNLs* can be observed in several angiosperms, such as most species of Ranunculales and Lamiales, and likely all monocots, suggesting that multiple and independent losses of *TNLs* have occurred throughout angiosperm evolution (Tarr and Alexander, 2009; Shao et al., 2016; Liu et al., 2021).

Different species are favored by different pathogens; therefore, *NLR* genes in different species evolved under different selection pressures and have distinct evolutionary patterns. For instance, *Brassicaceae* exhibit a “first expansion and then contraction” evolutionary pattern (Zhang et al., 2016), *Poaceae* manifest a “contraction” evolutionary pattern, and both *Fabaceae* and *Rosaceae* show a consistently expanding pattern (Shao et al., 2014; Jia et al., 2015). Moreover, it is not uncommon that the evolutionary patterns within the same family are distinct [e.g., potato, pepper, and tomato in *Solanaceae* (Qian et al., 2017); *Gastrodia elata*, *Apostasia shenzhenica*, *Phalaenopsis equestris*, and *Dendrobium catenatum* in *Orchidaceae* (Xue et al., 2020b); and *Xanthoceras sorbifolium*, *Acer yangbiense*, and *Dinnocarpus longan* in *Sapindaceae* (Zhou et al., 2020)].

Magnoliids (*Magnoliidae*), the third-largest group of angiosperms, are a clade of early diverging angiosperm lineages, encompassing over 10,000 living species that can be divided into four orders, namely, Piperales, Magnoliales, Laurales, and Canellales. Magnoliid plants are distributed throughout temperate and tropical zones of the world and are mainly found in the form of large trees, shrubs, vines, lianas, and occasionally herbs (Judd et al., 2002; Soltis et al., 2005). Traditionally, magnoliids have been classified as dicotyledonous plants, but the Angiosperm Phylogeny Group (APG) classification separates them from eudicots, and there exists a monophyletic branch with an unresolved phylogenetic position (Byng et al., 2016). Hence, magnoliids possess an important phylogenetic position that can be utilized for better comprehending the evolution of extant flowering plants.

**FIGURE 1:**
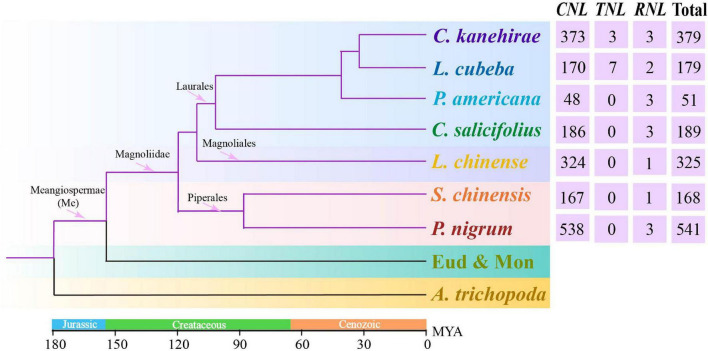
The phylogenetic tree of angiosperms, such as seven investigated magnoliids (*Liriodendron chinense* in Magnoliales, *Persea americana*, *Chimonanthus salicifolius*, *Litsea cubeba*, and *Cinnamomum kanehirae* in Laurales, *Piper nigrum* and *Saururus chinensis* in Piperales), was constructed according to the Angiosperm Phylogeny Group (APG) IV system (Byng et al., 2016). The divergence times at different nodes of angiosperms were combined from previous studies (Zhou et al., 2006, 2012; Nie et al., 2007; Magallón, 2010; Fiz-Palacios et al., 2011; Jiao et al., 2011).

Previous studies comprehensively clarified the evolutionary framework of angiosperm *NLR* genes exclusive to magnoliids (Shao et al., 2014, 2016; Zhang et al., 2016; Qian et al., 2017; Xue et al., 2020b; Zhou et al., 2020). In this study, we identified *NLR* genes in seven magnoliid genomes (Figure 1), performed a series of comprehensive analyses, and established an underlying framework of *NLR* gene evolution. This study uncovered the evolutionary features and patterns of the *NLR* gene family in magnoliids and investigated the mechanisms that molded these evolutionary changes. Collectively, our findings will serve as a fundamental resource for the mining of functional *R* genes in future investigations.

## Materials and Methods

### Data Source

The whole genomes of seven *Magnoliidae* species, *Liriodendron chinense* in Magnoliales, *P. americana*, *Chimonanthus salicifolius*, *Litsea cubeba*, and *Cinnamomum kanehirae* in Laurales, and *Piper nigrum* and *Saururus chinensis* in Piperales were used in this study. Genomic sequences and annotation files of *P. nigrum*, *C. salicifolius*, *L. chinense*, and *P. americana* were obtained from the cotton database^1^, Xuehui Huang Lab^2^, Hardwood Genomics Database^3^, and Comparative Genomics Database^4^, respectively. Genomic sequences and annotation files of *C. kanehirae* and *L. cubeba* were downloaded from the National Center for Biotechnology Information (NCBI) database^5^ (accession Nos. PRJNA477266 and PRJNA562049, respectively). Genomic sequences and annotation files of *S. chinensis* were from our own unpublished data, and all *S. chinensis NLR* genes have been deposited at the NCBI database (BioProject ID: PRJNA764779). These seven genomes all have a high quality according to two parameters, namely, N50 and BUSCO completeness (Supplementary Table 1).

### Identification and Classification of the *NLR* Genes

The *NLR* genes were identified in the seven magnoliid genomes as described previously (Shao et al., 2015). In brief, a two-step strategy was adopted to identify the *NLR* genes. The first step was to conduct simultaneously hidden Markov model searches (HMMsearch) and BLAST search using the amino acid sequence of the NB-ARC domain (Pfam accession number: PF00931) as a query to identify potential *NLR* genes. The threshold expectation value was set to 1.0 for the BLAST search. Then, the remaining candidate genes were merged, and the redundant hits were removed. For the sake of confirming the presence of the NBS domain, the remaining sequence hits were subjected to online Pfam analysis with an *E*-value of 10^––4^.^6^ All of the identified *NLR* genes were subjected to the conserved domain database (CDD) of NCBI^7^ using the default settings to determine whether they encoded CC, TIR, RPW8, or LRR domains.

### Distribution of *NLR* Genes in Different Chromosomes

To determine the distribution of *NLR* genes on chromosomes or scaffolds of magnoliid genomes, GFF3 annotation file was anatomized to extract the genomic locations of all identified *NLR* genes. To detect the organization of *NBS* genes on chromosomes or scaffolds, a sliding window size of 250 kb was used to identify the number of genes that appear in a cluster on a chromosome or a scaffold as described in the study by Ameline-Torregrosa et al. (2008). Based on this criterion, *NLR* genes were assigned to clustered loci and singleton loci, which were mapped along the chromosomes.

### Sequence Alignment and Conserved Motif Analysis

Amino acid sequences of the NBS domain were extracted from all identified *NLR* genes and used for multiple alignments using ClustalW integrated into MEGA 7.0 using default parameter settings (Kumar et al., 2016). Very short (<190 amino acids, less than two-thirds of a regular NBS domain) or very divergent sequences were removed to prevent interference with the alignments and subsequent phylogenetic inference. Then, the resulting alignments were manually corrected using MEGA 7.0 for further improvement (Kumar et al., 2016). The conserved protein motifs within the NBS domain of the three classes of *NLR* genes, along with the N-terminal domain plus the NBS domain of four subclasses of *CNL* genes, were analyzed by Multiple Expectation Maximization for Motif Elicitation (MEME) and WebLogo using default settings (Crooks et al., 2004; Bailey et al., 2006). Additionally, structural motif annotation was performed using the Pfam analysis^8^ and SMART tools^9^.

### Phylogenetic Analysis and Reconciliation of Gene Loss/Duplication Events

To explore the relationships of *NLR* genes in the magnoliids, a phylogenetic tree was constructed based on the aligned amino acid sequences of the conserved NBS domains. The phylogenetic analyses were conducted using IQ-TREE and the maximum-likelihood method (Nguyen et al., 2015). The best-fit model was estimated by ModelFinder (Kalyaanamoorthy et al., 2017). Branch support values were assessed with UFBoot2 tests (Minh et al., 2013). The scale bar indicated genetic distance. To trace ancestral *NLR* genes, representative genes from different angiosperm lineages were used as references. In our earlier study (Shao et al., 2016), we identified 55 and 56 ancestral *NLR* genes in eudicots and monocots, respectively, representing 55 and 56 ancestral *NLR* genes in the genomes of their ancestors. Representative genes were picked from each of the ancestral genes, based on the following conditions: genes should be located in internal nodes with shorter branch lengths in each ancestral gene, and genes from species, such as *Arabidopsis*, rice, tomato, and *Populus*, were preferred because the genomes of these model plants are better assembled and genes are better studied. These picked genes are good representatives of ancestral *NLR* genes in eudicots and monocots. In addition, all *NLRs* of *Amborella trichopoda*, which is the earliest-diverging angiosperm, were used as the representatives of basal angiosperms. These genes represent the ancestral *NLRs* in early diverging angiosperms, and they were used as a reference and analyzed together with magnoliid genes. Additionally, gene loss/duplication events were recovered by reconciling the phylogenetic tree of the *NLR* gene with the real magnoliid species tree using Notung software (Stolzer et al., 2012).

### Gene Duplication Type Determination

We employed the MCScanX package, which was developed and adopted by the Plant Genome Duplication Database (PGDD)^10^, to conduct the syntenic analysis of *NLR* genes among seven magnoliid genomes (Wang et al., 2012; Lee et al., 2013). In brief, pair-wise all-against-all BLAST was performed for the protein sequences within a genome. The obtained results and GFF annotation files were then subjected to MCScanX for microsynteny detection and determination of the gene duplication type (Wang et al., 2012). Microsynteny relationships were displayed using TBtools (Chen et al., 2020).

### Gene Expression Analysis

To analyze the expression of the *NLR* genes of *S. chinensis*, the RNA-seq data were generated by ourselves and checked with FastQC software to remove low-quality reads or adapter. Clean reads in each sample were mapped to the reference genome of *S. chinensis* using HISAT2 with default settings (Pertea et al., 2016). The mapping results were subjected to Cufflinks to assemble transcripts in each sample and then merged into one cohesive set using Cuffmerge. The expression of each gene was evaluated using Cuffdiff (Trapnell et al., 2012). All analyses by Cufflinks were performed with default settings. The genes with the reads per kilobase per million (RPKM) value larger than 100 were recognized as a high expression gene in the analysis.

## Results

### Identification and Classification of *NLR* Genes in the Seven Magnoliid Genomes

A total of 1,832 *NLR* genes were identified from seven magnoliid genomes. Specifically, 325 *NLR* genes from *L. chinense* were identified in Magnoliales; 51 from *P. americana*, 179 from *L. cubeba*, 189 from *C. salicifolius*, and 379 from *C. kanehirae* in Laurales; and 541 from *P. nigrum* and 168 from *S. chinensis* in Piperales (Figure 1 and Supplementary Table 2). The number of *CNL* genes was much greater than 100, while the number of *TNL* and *RNL* genes was less than 10. Thus, the *CNL* genes accounted for over 98.5% (1,804) of all *NLR* genes and heavily outnumbered other classes. In fact, *TNL* genes were completely absent from five genomes, suggesting that the magnoliids are the characteristic of the loss of *TNLs*. Moreover, the *RNL* subfamily was found in all seven magnoliid genomes but in extremely low numbers in each genome.

Notably, the number of *NLR* genes varied within the same order among the seven magnoliid genomes investigated in this study. In Laurales, *C. kanehirae* possessed the greatest number of *NLR* genes (379), which was more than 7-fold higher than the number observed in *P. americana*. In Piperales, there was a 3-fold difference in the gene numbers between *S. chinensis* and *P. nigrum.* Furthermore, our study found a peculiar type of *CNL* gene with an Rx_N domain at the N-terminus, which consisted of a total of ∼400 members within the seven magnoliid genomes and could be considered a specialized CC domain, wherein a CC adopts a 4-helical-bundle-fold (Hao et al., 2013). The Rx_N domain was named after the potato R protein, Rx, which functions against potato virus X (PVX; Sacco et al., 2007; Tameling and Baulcombe, 2007). It is ∼83 amino acids in length, which is slightly shorter than a normal CC domain. On the pathogenic stimulation, the Rx_N domain will undergo an intramolecular interaction with the NBS domain and an intermolecular combination with the Trp-Pro-Pro (WPP) domain of the Rx cofactor, i.e., Ran GTPase-activating protein 2 (RanGAP2), primarily through hydrophobic interactions and form a heterodimer that transfers signals downstream (Hao et al., 2013).

Of the seven magnoliid genomes surveyed, the intact *NLR* genes with all three domains (i.e., CC, TIR, and RPW8-NBS-LRR) accounted for only 12.5% (229) of the total, while other genes either lacked an N-terminal or LRR domain at the C-terminus or entirely lacked the domains at both termini. *S. chinensis* had the highest proportion of intact genes (24.4%), while *P. nigrum* had the lowest (2.8%). Other than genomic changes (e.g., recombination, fusion, and pseudogenization) that result in real truncated genes, other subjective factors, such as sequencing errors, assembly errors, and false annotations, also artificially elicit “truncated” genes.

Occasionally, *NLR* proteins fuse with other domains (e.g., WRKY). The unusually integrated domains (IDs) were identified and counted, which are presented in a pie chart (Supplementary Figure 1). A variety of unusual IDs were found, and the associated pentatricopeptide repeat (PPR) protein occupied a fairly large proportion (13.2%). Other IDs, comprising a negligible component, demonstrated that many kinds of domains were integrated.

### Distribution and Organization of *NLR* Genes in Magnoliid Genomes

The *NLR* genes were scattered unevenly among magnoliid chromosomes (Supplementary Figure 2). For instance, among the 24 chromosomes in *P. nigrum*, Chromosome 3 contained the most genes (135; 25.0%), while Chromosomes 6, 10, and 25 contained the least (only one gene in each chromosome) (Supplementary Figure 2A). Magnoliid *NLR* genes in clustered loci were much more abundant than in singleton loci, except for *P. americana*, where their ratio ranged from 1.4 to 9.6 among the seven genomes (Table 1). The majority of *NLR* genes in *P. americana* were organized into singletons. Moreover, the number of clusters with 10 or more *NLR* genes varied tremendously among these species. *L. cubeba* and *P. americana* had no *NLR* genes, and *P. nigrum* had the most with 18 loci. Furthermore, on average, a cluster in *P. nigrum* (8.5) and *C. kanehirae* (5.9) contained more *NLR* genes than clusters from the other five genomes. The two largest clusters were found in *P. nigrum* and *C. kanehirae*, containing 51 and 35 *NLR* genes, respectively (Table 1).

**TABLE 1:** Organization of nucleotide-binding site (NBS)-encoding genes in the seven magnoliid genomes.

Gene and Loci	*C. salicifolius*	*C. kanehirae*	*L. chinense*	*L. cubeba*	*P. nigrum*	*P. americana*	*S. chinensis*
**No. of chromosome-anchored *NLR* loci and genes**	84 (189)	105 (379)	170 (325)	118 (179)	85 (541)	44 (51)	8 5 (168)
**No. of singleton loci (No. of *NLR* genes)**	48 (48)	49 (49)	101 (101)	74 (74)	24 (24)	38 (38)	52 (52)
**No. of clustered loci (No. of *NLR* genes)**	36 (141)	56 (330)	69 (224)	44 (105)	61 (517)	6 (13)	33 (116)
**Clustered *NLR* genes/singleton *NLR* genes**	2.9	6.7	2.2	1.4	9.6	0.3	2.2
**Average (median) No. of *NLR* genes in clusters**	3.9 (3)	5.9 (3)	3.2 (2)	2.4 (2)	8.5 (5)	2.2 (2)	3.5 (3)
**No. of clusters with 10 or more *NLR* genes**	2	10	2	0	18	0	1
**No. of *NLR* genes in the largest cluster**	12	35	15	4	51	3	13

### Reconstructing the *NLR* Gene Phylogenies

**FIGURE 2:**
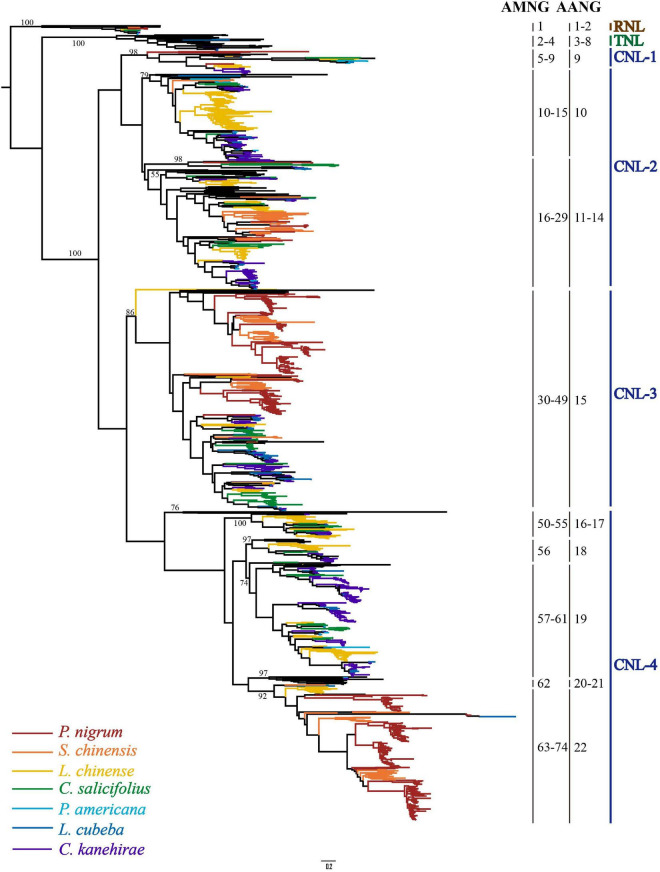
The phylogenetic relationships of *NLR* genes from seven magnoliid genomes. Predicted ancestral genes in the common ancestor of the seven magnoliids are indicated at the right of the phylogeny. *NLR* genes from different species are indicated with different colors in accordance with that of the species tree shown in Figure 1. Branch support values for three *NLR* classes (i.e., *CNL*, *TNL*, and *RNL*) and each gene are shown. The detailed phylogenetic tree is shown in Supplementary Figure 3.

To reconstruct the evolutionary relationship of magnoliid *NLR* genes, a phylogenetic tree was constructed based on the amino acid sequences of the conserved NBS domain and alignments, along with representative angiosperm *NLR* genes extracted from the study by Shao et al. (2016). The phylogenetic tree was composed of three monophyletic clades, namely, *RNL*, *TNL*, and *CNL*, with support values of 100%; many internal nodes had high (over 70%) support values. Compared to *TNLs* and *CNLs*, *RNL* genes had a relatively lower evolutionary rate, which was mirrored by the shorter branch lengths (Figure 2 and Supplementary Figure 3). In contrast, *CNL* genes exhibited an extremely active evolutionary pattern with far more gene duplications and losses, as well as faster evolutionary rates, which was reflected by the longer branch lengths (Figure 2). The *CNL* class could be further divided into four major subclasses. Every *CNL* subclass contained genes from all seven magnoliid species and representatives from eudicots and/or monocots, suggesting that the topology of the four subclasses should be stable at the whole angiosperm scale. However, the genes did not evenly fall into the four subclasses. Specifically, the *CNL-1* subclass contained the fewest with only 38 genes, while *CNL-2*, *-3*, and *-4* contained 346, 385, and 527 genes, respectively, which were at least 9-fold higher when compared to *CNL-1*. Genes with the Rx_N domain are mainly clustered into *CNL-3* (94.9%) and with a small number falling into *CNL-1* (1.9%) and *-2* (3.2%) (Supplementary Table 3). The distribution of Rx_N domains containing genes in the phylogenetic tree suggests that this specialized CC domain has multiple origins.

**FIGURE 3:**
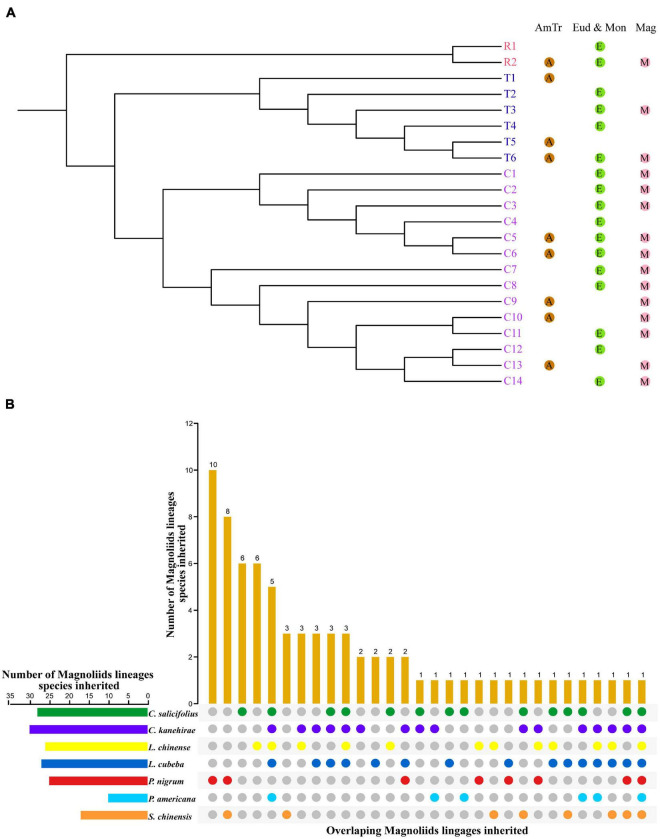
Inheritance of ancestral *NLR* genes. **(A)** Inheritance of ancestral angiosperm *NLR* genes in *Amborella trichopoda*, eudicots + monocots, and magnoliids. **(B)** Inheritance of ancestral magnoliid *NLR* genes in seven magnoliids.

Based on the reconstructed *NLR* gene phylogeny, 22 angiosperm ancestral *NLR* genes (AANGs), such as 14 *CNLs*, 6 *TNLs*, and 2 *RNLs*, were reconciled (Figure 2 and Supplementary Figure 4). Notably, not all ancestral *NLR* genes were preserved in all angiosperm clades, where only 4 out of the 22 AANGs (i.e., R2, T6, C5, and C6) were inherited by four early diverging angiosperm branches (i.e., magnoliids, eudicots, monocots, and *A. trichopoda*). No branch successfully maintained all 22 AANGs. Magnoliids lost seven AANGs (1 *RNL*, 2 *TNLs*, and 12 *CNLs*), eudicots and monocots together lost five genes, and *A. trichopoda* lost up to 10 AANGs (Figure 3A).

Reconciling the magnoliid *NLR* genes recovered 74 ancestral magnoliid *NLR* genes (AMNGs), such as 1 *RNL*, 3 *TNLs*, and 70 *CNLs*, in the common ancestor of all magnoliids, indicating that the 15 AANGs inherited by magnoliids intensively expanded to 74 genes before further taxa divergences in the magnoliid ancestor, among which, two *RNLs* dropped to one, two *TNLs* slightly expanded to three, and *CNLs* largely expanded from 12 to 70 genes. Further analysis revealed that 28, 30, 26, 27, 25, 10, and 17 of the 74 AMNGs were inherited by *C. salicifolius*, *C. kanehirae*, *L. chinense*, *L. cubeba*, *P. nigrum*, *P. americana*, and *S. chinensis*, respectively (Figure 3B and Supplementary Table 4). Notably, none of the genes were reserved in all seven magnoliid genomes, just as the magnoliid ancestor did not inherit all 22 AANGs due to independent gene losses that occurred throughout magnoliid evolution. The differential inheritance patterns suggest that AMNGs experienced distinct gene duplication/loss events. Both gene loss and duplication events resulted in the observed gene number variations among the seven different species.

### Conserved Motifs of the NBS Domain in Magnoliids

**FIGURE 4:**
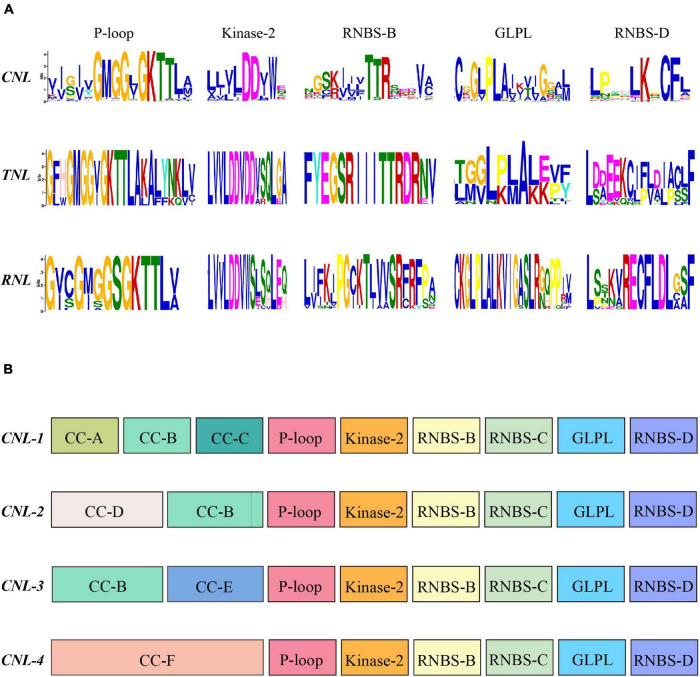
**(A)** Conserved motifs in the nucleotide-binding site (NBS) domain of the seven magnoliids. **(B)** Conserved motifs in the N-terminal domain and NBS domain of the four *CNL* subclasses. The amino acids of the 11 conserved motifs are extracted. Larger letters indicate higher frequency.

To explore the structural components and confirm the homology of all *NLR* genes in the magnoliid genomes, we searched for conserved motifs in the NBS domains *via* the MEME analysis (Figure 4A; DeYoung and Innes, 2006). From the N-terminus to the C-terminus, a total of five conserved motifs were identified, such as P-loop, Kinase-2, RNBS-B, GLPL, and RNBS-D. The P-loop, GLPL, and RNBS-B motifs exhibited high similarity among the three subclasses of *NLR* genes, suggesting that the homology of NBS domains with critical functions regulates immune responses. The other two motifs, especially RNBS-D, had distinct sequences among the three subclasses of *NLR* genes. These motifs can be utilized to distinguish the classes of magnoliid *NLR* genes without conducting phylogenetic analyses. The *CNL*s extensively duplicated throughout the evolution of magnoliids and had considerable sequence diversity, while *RNL*s and *TNL*s possessed remaining copies with hardly any detectable duplication events, which may explain their highly conserved motifs. Comparatively, the Kinase-2 motif had a conserved “DDVW” sequence in the *RNL* and *CNL* genes but frequently appeared as “DDVD” in the *TNL* genes, which is in agreement with previous angiosperm studies. However, in contrast to these previous studies, the “SR” sequence in the RNBS-B motif of *RNLs* was conserved, which may be a trait of magnoliids, while *CNL* and *TNL* proteins frequently appeared as “TTR” and “TTRD,” respectively (Shao et al., 2016).

Further analysis revealed that four *CNL* subclasses had unique, distinctive motifs at the N-terminal domain (e.g., CC-A, CC-C, and CC-D) (Figure 4B). Additionally, the more conserved amino acid sequence of “YDAED” at the CC-B motif shared three *CNL* subclasses (Supplementary Figure 5A). With regard to the NBS domain, although four *CNL* subclasses shared five conserved motifs, some amino acids at specific sites showed some discrepancies among the four subclasses and could be used as preliminary labels for classification, such as the “ELP” sequence in RNBS-D of the *CNL-1* subclass, “GSR” in RNBS-B of *CNL-3*, and “DD” in Kinase-2 of *CNL-4*. More importantly, in *CNL-3*, the RNBS-D motif had a conserved “CF” sequence but no “PED” sequence, while other subclasses appeared as “CFL” and had a “PED” sequence (Supplementary Figure 5B).

### Differential Losses and Frequent Duplication Events of *NLR* Genes During Magnoliid Evolution

**FIGURE 5:**
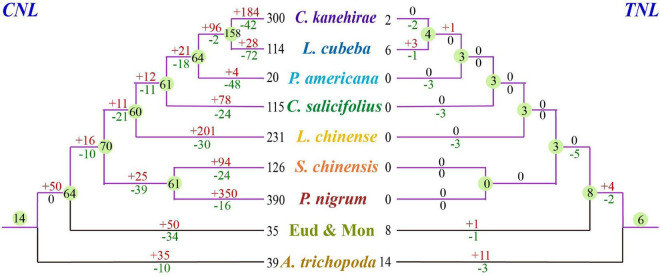
Loss and gain events of *NLR* genes across magnoliid evolution. Gene losses and gains are indicated by numbers with “–” or “+” on each branch. Detailed information for gain and loss events of *NLR* genes is shown in Supplementary Figure 4.

Based on the phylogenetic tree, we deduced that numerous independent gene duplication and loss events occurred at different stages of magnoliid evolution (Figure 5). The 74 ancestral *NLR* genes in the magnoliid ancestor should have undergone considerable complicated evolutionary processes to result in the current *NLR* genes observed in the seven magnoliid genomes of this study. The detailed evolutionary processes of the *TNL* and *CNL* genes were reconstructed, and species-specific gene duplication and loss events were detected, which reflected the diverse *NLR* gene number and the evolutionary patterns of the *NLR* genes in magnoliids (Figure 5).

**FIGURE 6:**
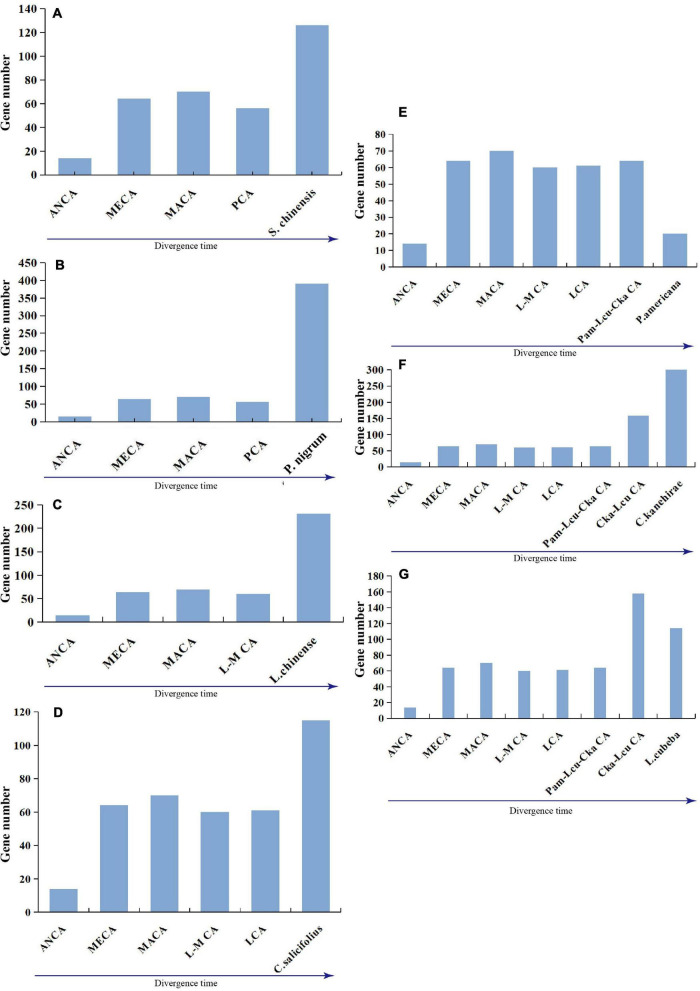
Evolutionary patterns of *NLR* genes in seven magnoliids: **(A)** *S. chinensis*, **(B)** *P. nigrum*, **(C)** *L. chinense*, **(D)** *C. salicifolius*, **(E)** *P. americana*, **(F)** *C. kanehirae*, and **(G)** *L. cubeba.* Pam-Lcu-Cka indicates the common ancestor of *P. americana*, *L. cubeba*, and *C. kanehirae*; Cka-Lcu indicates the common ancestor of *C. kanehirae* and *L. cubeba.*

Five species exhibited similar evolutionary patterns: a first expansion, followed by a slight contraction, and another expansion; among the two expansions, the most recent expansion appeared to be stronger (Figures 6A–D,F). Specifically, *P. nigrum* duplicated 350 genes and lost 16 genes, and the gene number accordingly sharply increased in the genome, which was likely due to a recent whole-genome duplication event (Figure 6B). Additionally, both *L. cubeba* and *P. americana* showed a two-times-repeated pattern of “expansion followed by contraction” (Figures 6E,G). In summary, the seven magnoliid genomes exhibited two dynamic and discrepant patterns of *NLR* gene evolution, and the discrepancy was dependent on whether a given taxon underwent a recent expansion/contraction.

Three types of *NLR* gene duplications, namely, local tandem, ectopic, and segmental duplications, have been defined (Leister, 2004), indicating that the tandem duplication events played major roles in *NLR* gene expansion in the seven magnoliid genomes. Ectopic and segmental duplication events were the main contributors to *NLR* gene expansion in *P. americana* and *P. nigrum* genomes (Table 2).

**TABLE 2:** Contributions of three duplication types in producing *NLR* genes during the evolution of magnoliids.

Different types of duplication	*C. salicifolius*	*C. kanehirae*	*L. chinense*	*P. americana*	*P. nigrum*	*L. cubeba*	*S. chinensis*
**Total No. of new duplicated genes**	189	379	325	51	541	179	168
**Local tandem duplication**	102 (54.0%)	334 (88.1%)	192 (59.1%)	5 (9.8%)	107 (19.8%)	82 (45.8%)	102 (60.7%)
**Ectopic duplication**	55 (29.1%)	45 (11.9%)	132 (40.6%)	44 (86.3%)	111 (20.5%)	78 (43.6%)	59 (35.1%)
**WGD or Segmental duplication**	32 (16.9%)	0	1 (0.3%)	2 (3.9%)	323 (59.7%)	19 (10.6%)	7 (4.2%)

### Expression Profile of *NLR* Genes in *S. chinensis*

**FIGURE 7:**
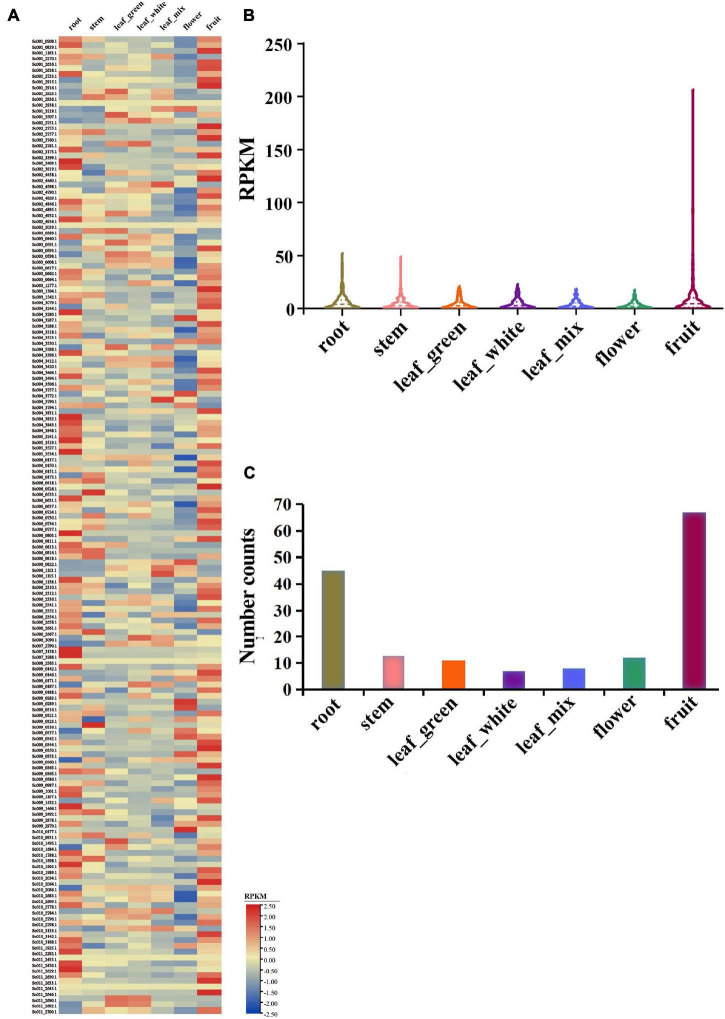
Expression pattern of *S. chinensis NLR* genes. **(A)** Heatmap of the expression of 168 *NLR* genes in seven different plant tissues. **(B)** Average expression of 168 *NLR* genes in the seven tissues. **(C)** Distribution of the top expression tissue for 168 *NLR* genes.

To obtain the expression profile of *NLR* genes in *S. chinensis*, the transcriptomes of seven *S. chinensis* tissues were analyzed. Results indicated that most *NLR* genes were only expressed at very low levels in all of the tissues [i.e., flowers, green leaves, white leaves, mixed color leaves (half green half white), roots, fruits, and stems]. Nonetheless, the expression of some particular genes reached 100 or up to 200 RPKM, showing unusually high expression levels (Figure 7 and Supplementary Table 5).

The average expression values of 168 genes were 6.4, 4.7, 4.3, 4.4, 3.7, 3.0, and 9.3 RPKM in the roots, stems, green leaves, white leaves, mixed color leaves, flowers, and fruits, respectively (Figure 7B). The roots and fruits had higher average *R* gene expression levels than the other tissues, which may be because the roots have greater direct physical interactions with soil microorganisms, providing easier access for infection, while the fruits may attract microorganisms and pathogens. The highest expression value of each gene was detected in the seven tissues. Results revealed that 168 genes were expressed in at least one tissue, 45 genes had the highest expression value in the roots, 13 in the stems, 11 in green leaves, 7 in white leaves, 8 in mixed color leaves, 12 in flowers, and 67 in the fruits (Figure 7C). Overall, the expression analysis indicated that *NLR* genes in *S. chinensis* were expressed at low levels with only some genes, showing high expression levels in specific tissues; these results are in accordance with the expressional trait of *R* genes, which usually remain silent but are highly expressed on exposure to pathogen stimuli.

## Discussion

### Dramatic Variations in *NLR* Gene Numbers Among Different Species

The discrepancy of *NLR* gene expression among angiosperms is extremely prominent. A recent study investigated 305 angiosperm genomes and discovered that the number of *NLR* genes per genome ranged from 5 to more than 2,000, except for one genome (*Utricularia gibba*), representing the only known land plant genome completely lacking in *NLR* genes (Liu et al., 2021). The *NLR* gene number varies greatly within the same family. Previous studies on *Fabaceae*, *Solanaceae*, *Poaceae*, and *Brassicaceae* identified 2- to 6-fold differences in *NLR* gene number among species in the same family, and up to a 20-fold difference was found in *Orchidaceae* (Luo et al., 2012; Shao et al., 2014; Zhang et al., 2016; Qian et al., 2017; Tirnaz et al., 2020; Xue et al., 2020b). As expected, among the seven magnoliid genomes examined in this study, the *NLR* gene numbers varied considerably. Our analyses covered three orders, and some taxa diverged more than 100 million years ago (One Thousand Plant Transcriptomes Initiative, 2019; Xue et al., 2020a; Yang et al., 2020). In Laurales, *P. americana* contained 51 *NLR* genes, while all the other three species had at least three times the number of genes. For example, *C. kanehirae* possessed 379 genes. The few *NLR* genes in *P. americana* may be related to its poor genome assembly and annotation (Rendón-Anaya et al., 2019); comparatively, all other magnoliids were better assembled.

According to the reconciled *NLR* gene gains and losses, *CNL* genes contributed much more to the current gene numbers and discrepancy among species than other classes (Figure 5). *CNL* genes (70/74) vastly outnumbered other classes in the common magnoliid ancestor and played an essential role in the discrepancy of *NLR* gene numbers we observed at present. Magnoliid *TNL* and *RNL* genes started with low copy numbers, and no follow-up vast expansions were detected, which led to minimal or overlooked effects on the whole *NLR* gene number variation. In actuality, the common magnoliid ancestor only inherited 14 *CNL* AANGs but quickly expanded up to 70 AMNGs before further species divergences. Among all four subclasses, *CNL-1*, *-2*, and *-4* expanded 4- to 5-fold more, while *CNL-3* genes increased up to 20-fold (from 1 to 20) and greatly contributed to the overall expansion. *CNL-3* also appeared to be a magnoliid-specific expanded subclass.

In terms of the underlying mechanism, tandem duplications largely accounted for most *NLR* gene expansions, which is consistent with other investigated angiosperm genes (e.g., legumes, *Brassicaceae*, orchids, *Sapindaceae*, and *Solanaceae*) (Shao et al., 2014; Zhang et al., 2016; Qian et al., 2017; Xue et al., 2020b; Zhou et al., 2020).

### Independent *TNL* Losses in Magnoliids

The hypothesis of *TNL* losses in the common monocot ancestor continues to receive increasing support from growing genomic data (Shao et al., 2016; Xue et al., 2020b). Recent studies reported the absence of *TNL* genes in monocots and certain eudicots, such as one basal eudicot (*Aquilegia coerulea*) and two Lamiales (*Sesamum indicum* and *Mimulus guttatus*) species (Collier et al., 2011; Shao et al., 2016). Therefore, these independent *TNL* losses may be the consequence of convergent evolution, which could be explained by inner factors, such as *TNL* genes starting with a few genes present in the common angiosperm ancestor that did not expand during the long-term evolution (Shao et al., 2016). Influences from outside particular habitats or lifestyles reduce the pathogenic threat, and some plants can afford the loss of several *R* genes (Liu et al., 2021).

The *TNL* gene loss has been increasingly observed in major angiosperm lineages, such as eudicots, monocots, and now in magnoliids, as well as more frequently among closely related taxa (e.g., within Lamiales) (Liu et al., 2021). Syntenic and phylogenetic evidence has shown that a whole-genome duplication event in the common angiosperm ancestor resulted in two *RNL* subclasses in angiosperms, namely, activated disease resistance 1 (*ADR1*) and N requirement gene 1 (*NRG1*; Shao et al., 2016; Van Ghelder et al., 2019). Growing evidence has also revealed that nearly all TNL proteins functionally rely on RNL proteins predominantly from the *NRG1* lineage to confer resistance (Qi et al., 2018; Castel et al., 2019; Wu et al., 2019; Saile et al., 2020). Interestingly, *RNL-NRG1* is synchronously absent with *TNLs*, supporting the hypothesis that *TNLs* rely on this indispensable downstream gene, i.e., *RNL-NRG1*, to transfer resistance signals (Collier et al., 2011).

## Data Availability Statement

The original contributions presented in the study are publicly available. This data can be found here: National Center for Biotechnology Information (NCBI) BioProject database under accession number PRJNA764779.

## Author Contributions

J-YW, J-YX, and YV designed the study and analyzed the data. J-YW wrote the manuscript. J-YX and YV participated in the revision of the manuscript. All authors read and approved the final manuscript.

## Conflict of Interest

The authors declare that the research was conducted in the absence of any commercial or financial relationships that could be construed as a potential conflict of interest.

## Publisher’s Note

All claims expressed in this article are solely those of the authors and do not necessarily represent those of their affiliated organizations, or those of the publisher, the editors and the reviewers. Any product that may be evaluated in this article, or claim that may be made by its manufacturer, is not guaranteed or endorsed by the publisher.

## Footnotes

1
http://cotton.hzau.edu.cn/EN/download.php

2
http://xhhuanglab.cn

3
https://hardwoodgenomics.org

4
https://genomevolution.org

5
https://www.ncbi.nlm.nih.gov/

6
http://pfam.sanger.ac.uk/

7
https://www.ncbi.nlm.nih.gov/Structure/cdd/wrpsb.cgi

8
http://pfam.janelia.org/

9
http://smart.embl-heidelberg.de/

10
http://chibba.agtec.uga.edu/duplication/
