# Supplementary material for: Evolution of NLR Resistance Genes in Magnoliids: Dramatic Expansions of CNLs and Multiple Losses of TNLs
Source: Front Plant Sci. 2021 Dec 21;12:777157. doi: 10.3389/fpls.2021.777157 (PMC8724549; doi:10.3389/fpls.2021.777157)
Supplement: Supplementary file 3 [file Data_Sheet_2.docx]

**Supporting information**

**Table S1** Assessment of seven magnoliids genome assembly.

**Table S2** A list of *NLR* genes identified from seven magnoliids.

**Table S3** A list of identified *CNL* genes with Rx_N domain at the beginning of the N-terminal CC domain.

**Table S4** Reservation of 74 ancestral magnoliids *NLR* genes in seven magnoliids. “+” indicates the presence of the ancestral gene, “-” indicates the absence of the ancestral gene.

**Table S5** .Expression of *NLR* genes in the seven *S. chinensis* tissues.

| Genomes of species | Genomes sequencing approaches | N50 | | Complete BUSCOs (C) |
| --- | --- | --- | --- | --- |
|  |  | contig (Mb) | scaffold (Mb) |  |
| *C. salicifolius* | PacBio + Illumina + Hi-C | 2.3 | —— | 95.10% |
| *C. kanehirae* | PacBio + Illumina + Hi-C | —— | 50.4 | 88.50% |
| *L. chinense* | PacBio + Illumina | 1.4 | 3.5 | 90.28% |
| *L. cubeba* | PacBio + 10× genomics + Hi-C | 0.6 | 1.8 | 89.20% |
| *P. americana* | PacBio + Illumina | 0.01 | 0.3 | 86.30% |
| *P. nigrum* | PacBio + Illumina + Hi-C | —— | 29.8 | 96.10% |
| *S. chinensis* | PacBio + Illumina | —— | 47.8 | 93% |

**Table S1** Assessment of seven magnoliids genome assembly.

**Table S3 A list of *CNL* genes with Rx_N domain at the beginning of the N-terminal CC domain.**

| Gene ID | CNL subclasses |
| --- | --- |
| Cs01g02891 | CNL-3 |
| Cs01g02899 | CNL-3 |
| Cs01g02900 | CNL-3 |
| Cs01g02952 | CNL-3 |
| Cs01g02954 | CNL-3 |
| Cs01g03299 | CNL-3 |
| Cs03g00372 | CNL-3 |
| Cs03g00382 | CNL-3 |
| Cs04g00081 | CNL-3 |
| Cs04g00085 | CNL-3 |
| Cs04g00125 | CNL-3 |
| Cs04g00129 | CNL-3 |
| Cs04g00169 | CNL-3 |
| Cs04g00696 | CNL-3 |
| Cs04g01116 | CNL-3 |
| Cs04g01118 | CNL-3 |
| Cs05g00276 | CNL-3 |
| Cs05g00318 | CNL-3 |
| Cs05g00323 | CNL-3 |
| Cs05g00324 | CNL-3 |
| Cs05g02493 | CNL-3 |
| Cs07g02305 | CNL-3 |
| Cs07g02306 | CNL-3 |
| Cs07g02307 | CNL-3 |
| Cs07g02309 | CNL-3 |
| Cs07g02310 | CNL-3 |
| Cs07g02311 | CNL-3 |
| Cs07g02512 | CNL-3 |
| Cs07g02757 | CNL-3 |
| Cs07g02758 | CNL-3 |
| Cs07g02760 | CNL-3 |
| Cs07g02761 | CNL-3 |
| Cs07g02790 | CNL-2 |
| Cs09g00177 | CNL-3 |
| Cs09g00191 | CNL-3 |
| Cs09g00342 | CNL-3 |
| Cs09g00351 | CNL-3 |
| Cs09g00382 | CNL-3 |
| Cs09g00383 | CNL-3 |
| Cs09g00402 | CNL-3 |
| Cs09g00423 | CNL-3 |
| Cs09g00424 | CNL-3 |
| Cs09g00441 | CNL-3 |
| Cs09g00459 | CNL-3 |
| Cs09g00462 | CNL-3 |
| Cs09g00465 | CNL-3 |
| Cs09g00467 | CNL-3 |
| Cs09g00468 | CNL-3 |
| Cs09g02755 | CNL-3 |
| Cs09g02764 | CNL-3 |
| evm.model.scaffold_1072.29 | CNL-3 |
| evm.model.scaffold_1080.2 | CNL-3 |
| evm.model.scaffold_110.91 | CNL-3 |
| evm.model.scaffold_110.95 | CNL-3 |
| evm.model.scaffold_1102.41 | CNL-3 |
| evm.model.scaffold_1404.46 | CNL-3 |
| evm.model.scaffold_148.437 | CNL-3 |
| evm.model.scaffold_148.453 | CNL-3 |
| evm.model.scaffold_154.53 | CNL-3 |
| evm.model.scaffold_156.217 | CNL-3 |
| evm.model.scaffold_160.34 | CNL-3 |
| evm.model.scaffold_180.116 | CNL-3 |
| evm.model.scaffold_180.118 | CNL-3 |
| evm.model.scaffold_180.121 | CNL-3 |
| evm.model.scaffold_180.93 | CNL-3 |
| evm.model.scaffold_210.102 | CNL-3 |
| evm.model.scaffold_210.112 | CNL-3 |
| evm.model.scaffold_210.132 | CNL-3 |
| evm.model.scaffold_210.138 | CNL-3 |
| evm.model.scaffold_210.41 | CNL-3 |
| evm.model.scaffold_211.29 | CNL-2 |
| evm.model.scaffold_211.33 | CNL-2 |
| evm.model.scaffold_211.50 | CNL-2 |
| evm.model.scaffold_223.39 | CNL-2 |
| evm.model.scaffold_255.23 | CNL-2 |
| evm.model.scaffold_298.33 | CNL-3 |
| evm.model.scaffold_298.35 | CNL-3 |
| evm.model.scaffold_298.39 | CNL-3 |
| evm.model.scaffold_298.62 | CNL-3 |
| evm.model.scaffold_298.66 | CNL-3 |
| evm.model.scaffold_298.71 | CNL-3 |
| evm.model.scaffold_357.94 | CNL-3 |
| evm.model.scaffold_440.58 | CNL-3 |
| evm.model.scaffold_52.13 | CNL-3 |
| evm.model.scaffold_607.4 | CNL-3 |
| evm.model.scaffold_620.55 | CNL-3 |
| evm.model.scaffold_666.48 | CNL-3 |
| evm.model.scaffold_666.60 | CNL-3 |
| evm.model.scaffold_725.2 | CNL-3 |
| evm.model.scaffold_725.5 | CNL-3 |
| evm.model.scaffold_784.1 | CNL-3 |
| evm.model.scaffold_860.61 | CNL-3 |
| evm.model.scaffold_860.62 | CNL-3 |
| evm.model.scaffold_923.58 | CNL-3 |
| Lchi05889 | CNL-3 |
| Lchi19554 | CNL-3 |
| Lchi20562 | CNL-3 |
| Lchi23057 | CNL-3 |
| Lchi23058 | CNL-3 |
| Lchi23067 | CNL-3 |
| Lchi24401 | CNL-3 |
| Lchi31278 | CNL-3 |
| Lchi31279 | CNL-3 |
| Lchi31283 | CNL-3 |
| Lchi31899 | CNL-3 |
| Lchi32864 | CNL-3 |
| Lchi34869 | CNL-3 |
| Pn1.3498 | CNL-2 |
| Pn1.3550 | CNL-2 |
| Pn11.1802 | CNL-3 |
| Pn11.1803 | CNL-3 |
| Pn11.1805 | CNL-3 |
| Pn11.1812 | CNL-3 |
| Pn11.1816 | CNL-3 |
| Pn11.1821 | CNL-3 |
| Pn11.1823 | CNL-3 |
| Pn11.1825 | CNL-3 |
| Pn11.1828 | CNL-3 |
| Pn11.1836 | CNL-3 |
| Pn11.1839 | CNL-3 |
| Pn11.1840 | CNL-3 |
| Pn11.1843 | CNL-3 |
| Pn11.1846 | CNL-3 |
| Pn11.1852 | CNL-3 |
| Pn11.1911 | CNL-3 |
| Pn11.1913 | CNL-3 |
| Pn11.1919 | CNL-3 |
| Pn11.1922 | CNL-3 |
| Pn11.1926 | CNL-3 |
| Pn11.1929 | CNL-3 |
| Pn11.1931 | CNL-3 |
| Pn11.1935 | CNL-3 |
| Pn11.1942 | CNL-3 |
| Pn11.1943 | CNL-3 |
| Pn11.1946 | CNL-3 |
| Pn11.1950 | CNL-3 |
| Pn11.1951 | CNL-3 |
| Pn11.1957 | CNL-3 |
| Pn11.1961 | CNL-3 |
| Pn12.1089 | CNL-3 |
| Pn12.1091 | CNL-3 |
| Pn12.1095 | CNL-3 |
| Pn12.1098 | CNL-3 |
| Pn12.1101 | CNL-3 |
| Pn13.1403 | CNL-3 |
| Pn16.107 | CNL-3 |
| Pn16.1366 | CNL-3 |
| Pn16.1370 | CNL-3 |
| Pn16.1495 | CNL-3 |
| Pn16.1656 | CNL-3 |
| Pn16.435 | CNL-3 |
| Pn16.440 | CNL-3 |
| Pn16.446 | CNL-3 |
| Pn16.450 | CNL-3 |
| Pn16.500 | CNL-3 |
| Pn16.505 | CNL-3 |
| Pn16.513 | CNL-3 |
| Pn16.519 | CNL-3 |
| Pn16.588 | CNL-3 |
| Pn16.591 | CNL-3 |
| Pn16.593 | CNL-3 |
| Pn16.600 | CNL-3 |
| Pn16.609 | CNL-3 |
| Pn16.628 | CNL-3 |
| Pn16.631 | CNL-3 |
| Pn16.636 | CNL-3 |
| Pn16.638 | CNL-3 |
| Pn16.645 | CNL-3 |
| Pn16.648 | CNL-3 |
| Pn16.651 | CNL-3 |
| Pn16.675 | CNL-3 |
| Pn16.678 | CNL-3 |
| Pn16.685 | CNL-3 |
| Pn16.690 | CNL-3 |
| Pn16.697 | CNL-3 |
| Pn16.707 | CNL-3 |
| Pn16.710 | CNL-3 |
| Pn16.719 | CNL-3 |
| Pn16.722 | CNL-3 |
| Pn16.772 | CNL-3 |
| Pn16.780 | CNL-3 |
| Pn16.786 | CNL-3 |
| Pn16.811 | CNL-3 |
| Pn20.1069 | CNL-3 |
| Pn20.1077 | CNL-3 |
| Pn20.1085 | CNL-3 |
| Pn20.1099 | CNL-3 |
| Pn20.1114 | CNL-3 |
| Pn22.924 | CNL-3 |
| Pn23.28 | CNL-3 |
| Pn23.87 | CNL-3 |
| Pn24.521 | CNL-3 |
| Pn30.75 | CNL-3 |
| Pn30.78 | CNL-3 |
| Pn30.80 | CNL-3 |
| Pn30.83 | CNL-3 |
| Pn30.85 | CNL-3 |
| Pn30.90 | CNL-3 |
| Pn30.91 | CNL-3 |
| Pn30.92 | CNL-3 |
| Pn30.98 | CNL-3 |
| Pn4.1300 | CNL-3 |
| Pn42.118 | CNL-3 |
| Pn42.122 | CNL-3 |
| Pn42.124 | CNL-3 |
| Pn42.128 | CNL-3 |
| Pn42.129 | CNL-3 |
| Pn42.130 | CNL-3 |
| Pn42.134 | CNL-3 |
| Pn42.136 | CNL-3 |
| Pn42.141 | CNL-3 |
| Pn50.32 | CNL-3 |
| Pn6.1680 | CNL-3 |
| Pn6.3104 | CNL-3 |
| Pn6.759 | CNL-3 |
| Pn6.777 | CNL-3 |
| Pn8.1630 | CNL-3 |
| Pn8.2650 | CNL-3 |
| Pn9.145 | CNL-3 |
| RWR72548.1 | CNL-3 |
| RWR72576.1 | CNL-3 |
| RWR72579.1 | CNL-3 |
| RWR72600.1 | CNL-3 |
| RWR74765.1 | CNL-3 |
| RWR74854.1 | CNL-3 |
| RWR74856.1 | CNL-3 |
| RWR74859.1 | CNL-3 |
| RWR74860.1 | CNL-3 |
| RWR74892.1 | CNL-3 |
| RWR74913.1 | CNL-3 |
| RWR74927.1 | CNL-3 |
| RWR74928.1 | CNL-3 |
| RWR74935.1 | CNL-3 |
| RWR74943.1 | CNL-3 |
| RWR74944.1 | CNL-3 |
| RWR75758.1 | CNL-3 |
| RWR75760.1 | CNL-3 |
| RWR75771.1 | CNL-3 |
| RWR75781.1 | CNL-3 |
| RWR75836.1 | CNL-3 |
| RWR75840.1 | CNL-3 |
| RWR75848.1 | CNL-3 |
| RWR75888.1 | CNL-3 |
| RWR75941.1 | CNL-3 |
| RWR76747.1 | CNL-3 |
| RWR76748.1 | CNL-3 |
| RWR76749.1 | CNL-3 |
| RWR79700.1 | CNL-3 |
| RWR79701.1 | CNL-3 |
| RWR79715.1 | CNL-3 |
| RWR79767.1 | CNL-3 |
| RWR79791.1 | CNL-3 |
| RWR84015.1 | CNL-1 |
| RWR84772.1 | CNL-2 |
| RWR84824.1 | CNL-2 |
| RWR85657.1 | CNL-1 |
| RWR85667.1 | CNL-1 |
| RWR85668.1 | CNL-1 |
| RWR85669.1 | CNL-1 |
| RWR86307.1 | CNL-3 |
| RWR90852.1 | CNL-3 |
| RWR91732.1 | CNL-3 |
| RWR91733.1 | CNL-3 |
| RWR91737.1 | CNL-3 |
| RWR91775.1 | CNL-3 |
| RWR91777.1 | CNL-3 |
| RWR91855.1 | CNL-3 |
| RWR91859.1 | CNL-3 |
| RWR91864.1 | CNL-3 |
| RWR91871.1 | CNL-3 |
| RWR91943.1 | CNL-3 |
| RWR93015.1 | CNL-3 |
| Sc001_2733.1 | CNL-3 |
| Sc002_4460.1 | CNL-3 |
| Sc002_4598.1 | CNL-3 |
| Sc002_4790.1 | CNL-3 |
| Sc002_4829.1 | CNL-3 |
| Sc002_4846.1 | CNL-3 |
| Sc002_4932.1 | CNL-3 |
| Sc002_4934.1 | CNL-3 |
| Sc003_0389.1 | CNL-3 |
| Sc003_0440.1 | CNL-3 |
| Sc003_0593.1 | CNL-3 |
| Sc003_0598.1 | CNL-3 |
| Sc003_0608.1 | CNL-3 |
| Sc003_0617.1 | CNL-3 |
| Sc003_1277.1 | CNL-3 |
| Sc003_1542.1 | CNL-1 |
| Sc004_3079.1 | CNL-3 |
| Sc004_3280.1 | CNL-3 |
| Sc004_3287.1 | CNL-3 |
| Sc004_3318.1 | CNL-3 |
| Sc004_3330.1 | CNL-3 |
| Sc004_3388.1 | CNL-3 |
| Sc004_3412.1 | CNL-3 |
| Sc004_3420.1 | CNL-3 |
| Sc004_3506.1 | CNL-3 |
| Sc004_3831.1 | CNL-3 |
| Sc004_3832.1 | CNL-3 |
| Sc006_0137.1 | CNL-3 |
| Sc006_1138.1 | CNL-3 |
| Sc009_1107.1 | CNL-3 |
| Sc010_1788.1 | CNL-3 |
| Sc010_1898.1 | CNL-3 |
| Sc010_1989.1 | CNL-3 |
| Sc010_2064.1 | CNL-3 |
| Sc010_2086.1 | CNL-3 |
| Sc010_2699.1 | CNL-3 |
| Sc010_2778.1 | CNL-3 |
| Sc010_2784.1 | CNL-3 |
| Sc010_2796.1 | CNL-3 |
| Sc010_2798.1 | CNL-3 |
| Sc010_3188.1 | CNL-3 |
| Sc011_2292.1 | CNL-3 |

**Table S4** Reservation of 74 ancestral magnoliids *NLR* genes in seven magnoliids. “+” indicates the presence of the ancestral genee, “-” indicates the absence of the ancestral gene.

| **Gene** | ***C. salicifolius*** | ***C. kanehirae*** | ***L. chinense*** | ***L. cubeba*** | ***P. nigrum*** | ***P. americana*** | ***S. chinensis*** |
| --- | --- | --- | --- | --- | --- | --- | --- |
| R1 | + | + | + | + | + | + | + |
| T1 |  |  | + |  |  |  |  |
| T2 |  | + |  |  |  |  |  |
| T3 |  | + |  | + |  |  |  |
| C1 |  |  |  |  | + |  |  |
| C2 |  | + | + | + |  |  | + |
| C3 | + |  |  |  |  |  |  |
| C4 | + | + | + | + |  | + |  |
| C5 |  | + | + |  | + |  |  |
| C6 |  |  |  | + |  |  |  |
| C7 | + |  |  |  |  | + |  |
| C8 |  |  |  |  |  |  | + |
| C9 | + | + | + | + |  | + |  |
| C10 |  |  | + |  |  |  |  |
| C11 | + | + |  | + |  | + |  |
| C12 |  |  |  |  | + |  |  |
| C13 | + | + |  | + |  |  |  |
| C14 | + |  |  |  |  |  |  |
| C15 | + | + |  |  |  |  |  |
| C16 |  | + | + |  |  |  |  |
| C17 |  |  | + |  |  |  |  |
| C18 |  |  |  |  | + |  | + |
| C19 | + | + |  | + |  |  |  |
| C20 | + |  | + |  |  |  |  |
| C21 | + |  | + |  |  |  |  |
| C22 |  |  |  |  | + |  | + |
| C23 |  |  |  |  | + |  | + |
| C24 | + | + | + | + |  | + |  |
| C25 |  | + | + | + |  | + |  |
| C26 |  |  | + |  |  |  |  |
| C27 |  |  |  |  | + |  | + |
| C28 |  |  |  |  |  |  | + |
| C29 |  |  |  |  | + |  |  |
| C30 |  |  | + |  | + |  |  |
| C31 |  |  |  |  | + |  | + |
| C32 |  | + |  | + | + |  |  |
| C33 | + |  | + | + |  |  |  |
| C34 | + |  |  | + |  |  |  |
| C35 | + | + |  |  |  |  | + |
| C36 |  |  |  |  | + |  | + |
| C37 | + |  |  |  |  |  |  |
| C38 |  | + |  | + |  |  |  |
| C39 | + |  |  |  |  |  |  |
| C40 |  | + |  |  |  |  |  |
| C41 |  | + |  | + |  |  |  |
| C42 |  | + | + |  |  |  |  |
| C43 | + | + |  | + | + |  | + |
| C44 |  | + | + |  |  |  |  |
| C45 | + | + |  | + |  |  |  |
| C46 | + |  |  |  |  |  |  |
| C47 |  | + |  | + | + |  |  |
| C48 |  |  | + |  |  |  |  |
| C49 | + | + | + | + |  |  |  |
| C50 |  |  | + |  |  |  | + |
| C51 | + | + | + | + |  |  |  |
| C52 | + | + | + | + |  | + |  |
| C53 | + |  |  |  |  |  |  |
| C54 |  |  |  | + |  |  |  |
| C55 |  |  |  |  | + |  |  |
| C56 | + | + | + | + |  |  |  |
| C57 | + | + | + | + |  | + |  |
| C58 |  | + |  |  |  | + |  |
| C59 | + |  |  | + |  |  | + |
| C60 |  |  | + |  |  |  |  |
| C61 |  |  |  |  | + |  |  |
| C62 |  |  |  |  | + |  |  |
| C63 |  |  |  |  | + |  | + |
| C64 |  |  |  |  |  |  | + |
| C65 |  |  |  |  | + |  |  |
| C66 |  |  |  |  | + |  |  |
| C67 |  |  |  |  | + |  |  |
| C68 |  |  |  |  | + |  |  |
| C69 |  |  |  | + | + |  |  |
| C70 |  |  |  |  | + |  | + |

**Table S5** Expression of *NLR* genes in the seven *S. chinensis* tissues.

| gene | root | stem | leaf_green | leaf_white | leaf_mix | flower | fruit |
| --- | --- | --- | --- | --- | --- | --- | --- |
| Sc001_0208.1 | 15.66769797 | 11.21983922 | 5.52880576 | 5.939979105 | 4.650757112 | 1.442960828 | 15.49262038 |
| Sc001_0839.1 | 2.221718089 | 0.607362812 | 0.960521827 | 0.639306078 | 0.950747068 | 0.079238314 | 1.55694103 |
| Sc001_1163.1 | 2.735061227 | 1.733300228 | 1.981831869 | 1.586937256 | 1.126591448 | 1.09296831 | 5.162597805 |
| Sc001_2270.1 | 19.06163011 | 18.35230113 | 15.16137009 | 16.3455511 | 18.85907493 | 12.88372863 | 14.37725802 |
| Sc001_2636.1 | 35.84856546 | 11.67000843 | 15.28706461 | 20.51225196 | 13.35226972 | 7.042744602 | 50.64909081 |
| Sc001_2638.1 | 3.379107156 | 1.393845656 | 4.219278722 | 3.851857193 | 1.771228712 | 0.354598036 | 8.228215357 |
| Sc001_2733.1 | 42.29412811 | 24.48506968 | 19.31302814 | 18.65966148 | 17.65725694 | 12.01486655 | 32.12104256 |
| Sc001_2915.1 | 0.08921246 | 13.15570971 | 16.53458619 | 16.17626415 | 13.10207058 | 5.07098103 | 37.16815653 |
| Sc001_2916.1 | 0 | 0.420950711 | 0.510715188 | 0.19840313 | 0.24619943 | 0.237979953 | 2.271214693 |
| Sc001_2923.1 | 0 | 2.176897625 | 3.829281586 | 1.475315219 | 2.512780202 | 0 | 0.753710536 |
| Sc001_2936.1 | 0 | 0.43702869 | 0.33388115 | 0.131678086 | 0.341031191 | 0 | 0 |
| Sc001_2938.1 | 0 | 0 | 0 | 0 | 0 | 0 | 0 |
| Sc001_3219.1 | 0.253788364 | 0.116648992 | 0.929094322 | 0.796341794 | 1.578262519 | 1.813521927 | 0.703416599 |
| Sc001_3507.1 | 0.443701289 | 0.317238217 | 4.725654767 | 2.395289312 | 1.942332788 | 0.201765612 | 2.945027748 |
| Sc002_2751.1 | 0 | 2.392645135 | 16.34296066 | 23.38161199 | 17.03780053 | 9.342764688 | 5.403924615 |
| Sc002_2755.1 | 0.085882906 | 0 | 0.029979666 | 0.013112771 | 0 | 0 | 0.876984817 |
| Sc002_2757.1 | 11.60693316 | 14.43059129 | 5.144900572 | 6.370979149 | 4.096131627 | 3.245026638 | 12.86194607 |
| Sc002_2760.1 | 0.343531623 | 0.092107035 | 0.096750726 | 0.098132946 | 0.057818249 | 0.117161371 | 0.876984817 |
| Sc002_2761.1 | 0.278431769 | 0 | 0.460249878 | 0.559643077 | 0.121669624 | 0.12661211 | 0 |
| Sc002_3175.1 | 4.462154321 | 0.929230437 | 0.773626412 | 1.579710117 | 1.004874342 | 2.797384503 | 2.786976169 |
| Sc002_3399.1 | 23.70625564 | 49.21884042 | 21.55382234 | 18.68152179 | 15.27652795 | 8.134371363 | 91.24331082 |
| Sc002_3409.1 | 2.142267293 | 0 | 0 | 0 | 0 | 0 | 0 |
| Sc002_3819.1 | 0.899846984 | 0 | 0.272584062 | 0.233084883 | 0.155477707 | 0.430726427 | 0.386892576 |
| Sc002_4458.1 | 9.965077821 | 7.5821845 | 11.03570443 | 10.44641931 | 9.213173426 | 8.634248553 | 11.96337103 |
| Sc002_4460.1 | 3.642519698 | 1.444870363 | 1.233696123 | 1.787022592 | 1.401338942 | 3.312747719 | 11.92285468 |
| Sc002_4598.1 | 0 | 0 | 0.05442514 | 0.103784485 | 0.159572307 | 0.045847942 | 0 |
| Sc002_4790.1 | 11.39888847 | 7.937385936 | 12.91140178 | 12.94833888 | 11.97471274 | 6.370385655 | 14.20400608 |
| Sc002_4829.1 | 9.870699932 | 9.276072243 | 8.691090611 | 9.542915693 | 6.07580772 | 4.293379253 | 15.74269155 |
| Sc002_4846.1 | 4.782439087 | 3.173358719 | 2.267173749 | 2.561469765 | 1.268652464 | 0.68840463 | 3.12687124 |
| Sc002_4895.1 | 9.814111412 | 7.371420979 | 6.749189997 | 7.755394092 | 5.957534327 | 3.358762512 | 10.4056855 |
| Sc002_4932.1 | 8.749127851 | 10.10221589 | 12.24360478 | 10.64535994 | 7.982795282 | 7.011945731 | 11.26174004 |
| Sc002_4934.1 | 11.80970977 | 6.711383983 | 3.041923592 | 2.100417512 | 1.833653997 | 2.276525206 | 6.390154548 |
| Sc002_5039.1 | 0 | 0 | 0 | 0 | 0 | 0 | 0 |
| Sc003_0389.1 | 0.409936595 | 2.951906361 | 3.265909089 | 0.965451019 | 0.542206618 | 1.864116919 | 0.239201547 |
| Sc003_0440.1 | 1.529490607 | 0.757078272 | 0.730696299 | 1.256697702 | 1.064392872 | 0.214003025 | 0.480561304 |
| Sc003_0591.1 | 1.949484897 | 8.223689368 | 16.49414266 | 11.48986693 | 9.979585572 | 1.063794107 | 8.095502206 |
| Sc003_0593.1 | 0.410059367 | 0.967510056 | 0.592928923 | 0.699977356 | 0.431959213 | 0.298348033 | 1.423675458 |
| Sc003_0598.1 | 0.844309287 | 1.43370563 | 2.461371777 | 2.208272791 | 1.577069429 | 1.087816636 | 1.868009207 |
| Sc003_0608.1 | 5.925478458 | 5.923024127 | 9.816507574 | 9.305307057 | 8.844446276 | 1.88354133 | 7.63686117 |
| Sc003_0617.1 | 14.21278784 | 10.21121002 | 18.14405858 | 16.98262452 | 18.11955466 | 5.019824043 | 20.00855554 |
| Sc003_0662.1 | 3.360499737 | 2.310283823 | 1.311865551 | 1.03298162 | 1.468477318 | 0.666107984 | 3.123582322 |
| Sc003_0664.1 | 7.062635634 | 4.721754286 | 3.172381284 | 4.516883385 | 2.816567183 | 4.92169325 | 7.305602245 |
| Sc003_1277.1 | 1.891281447 | 1.174306098 | 1.030804967 | 2.152946604 | 1.749411911 | 0.362117078 | 2.032908826 |
| Sc003_1304.1 | 52.60265446 | 41.64925845 | 17.32506681 | 12.40134754 | 6.906683455 | 5.915274755 | 207.0051353 |
| Sc003_1542.1 | 4.866782795 | 5.070361293 | 2.430500573 | 2.830058498 | 1.895046833 | 2.950782231 | 5.442968862 |
| Sc004_3079.1 | 6.803419758 | 3.977179125 | 2.648629224 | 3.303136367 | 2.449966019 | 3.169814942 | 7.516691251 |
| Sc004_3244.1 | 2.404001674 | 2.812609222 | 3.085820228 | 2.697841301 | 2.875176433 | 2.782637099 | 3.570651033 |
| Sc004_3280.1 | 7.58305908 | 2.296269331 | 3.137917278 | 2.966349161 | 3.181853547 | 2.271799501 | 4.099429029 |
| Sc004_3287.1 | 4.863026633 | 4.291202242 | 4.18540026 | 4.53377089 | 4.090897184 | 6.326222389 | 4.934402339 |
| Sc004_3288.1 | 32.75734469 | 18.07281721 | 15.41309414 | 15.17073593 | 10.39976723 | 12.90191314 | 45.82891363 |
| Sc004_3318.1 | 4.870568744 | 3.327101842 | 4.412084878 | 3.814478399 | 4.532837954 | 1.636418849 | 5.005211762 |
| Sc004_3323.1 | 7.595669091 | 2.785728148 | 4.477387169 | 5.817092678 | 4.988121059 | 2.970799035 | 7.233801325 |
| Sc004_3330.1 | 1.063998104 | 1.521477941 | 0.847157977 | 0.831659318 | 0.696162753 | 1.903084471 | 2.028116838 |
| Sc004_3388.1 | 0.717478547 | 1.135892909 | 1.676542427 | 1.119764844 | 1.499416632 | 1.087537207 | 0.907085694 |
| Sc004_3396.1 | 5.82532521 | 3.453902285 | 2.953510992 | 2.230974968 | 2.161271637 | 0.904525498 | 3.095140605 |
| Sc004_3412.1 | 5.563940425 | 4.429240916 | 7.028804918 | 6.735211997 | 6.919792702 | 2.086686387 | 5.769422075 |
| Sc004_3420.1 | 5.418314539 | 3.924701447 | 7.001830847 | 6.985773679 | 8.117561689 | 2.863784135 | 6.343958082 |
| Sc004_3464.1 | 5.70257487 | 4.58689004 | 3.659665458 | 4.166313086 | 3.780878494 | 1.996724653 | 8.06503235 |
| Sc004_3494.1 | 3.124365812 | 1.46597311 | 1.020078349 | 1.568262158 | 1.123878891 | 1.183960163 | 2.392811487 |
| Sc004_3506.1 | 5.236735846 | 4.914222209 | 5.808290191 | 7.352286775 | 6.83538708 | 2.948296655 | 9.135211927 |
| Sc004_3757.1 | 1.496380575 | 1.14630476 | 0.712715036 | 0.887274449 | 0.583243324 | 0.097207658 | 1.528014257 |
| Sc004_3772.1 | 1.434387666 | 2.703750828 | 1.672151531 | 3.254502809 | 2.477410564 | 4.704202606 | 2.263643978 |
| Sc004_3790.1 | 0.115227286 | 0.061789034 | 0.251686082 | 0.143253619 | 0.654578991 | 0.261988239 | 0 |
| Sc004_3794.1 | 0.131568375 | 0.141103434 | 0.023673716 | 0.051839964 | 0.041442289 | 0.159542614 | 0 |
| Sc004_3831.1 | 0 | 0 | 0.116728729 | 0.21944902 | 0.217994686 | 0.086987782 | 0.488345458 |
| Sc004_3832.1 | 0.417152811 | 0 | 0.028008745 | 0.043422345 | 0 | 0.042154032 | 0.094660328 |
| Sc004_3843.1 | 9.887861994 | 3.658306384 | 1.782772266 | 2.50067134 | 1.849082834 | 3.573508018 | 6.305046529 |
| Sc004_3848.1 | 7.739933444 | 5.27651817 | 2.707786258 | 3.991252173 | 2.607619481 | 4.856506531 | 6.739466913 |
| Sc005_3241.1 | 9.018720477 | 4.381481723 | 5.531072865 | 6.116898778 | 4.755655557 | 2.593864251 | 7.635124862 |
| Sc005_3519.1 | 10.88125778 | 2.693041657 | 0.140753206 | 0.25248745 | 0.160260553 | 1.802939336 | 3.013993372 |
| Sc005_3527.1 | 17.39124176 | 16.23466577 | 14.73568784 | 15.39988914 | 13.54804339 | 16.86087964 | 18.80098163 |
| Sc005_3534.1 | 2.555113164 | 0.188985379 | 0.117343522 | 0.057463918 | 0 | 0 | 0 |
| Sc006_0137.1 | 1.316790921 | 1.647592032 | 1.766250545 | 1.769013326 | 1.684736639 | 0.673636801 | 1.652771983 |
| Sc006_0150.1 | 4.599053937 | 4.51637581 | 3.764204916 | 6.380202015 | 4.383215018 | 4.585833235 | 7.921433683 |
| Sc006_0151.1 | 5.458557713 | 4.488183011 | 4.838189904 | 5.085567835 | 4.521136107 | 2.151228083 | 6.502944395 |
| Sc006_0173.1 | 0.37857739 | 1.533829725 | 0.858188359 | 0.749576553 | 0.818765509 | 0.841630176 | 1.718135846 |
| Sc006_0418.1 | 4.34662929 | 5.226686554 | 2.680790971 | 2.997861086 | 1.611610435 | 1.557286322 | 3.362513336 |
| Sc006_0526.1 | 12.985555 | 9.847713146 | 5.67847437 | 7.061503404 | 4.623726051 | 7.60973951 | 41.05628783 |
| Sc006_0533.1 | 0.618143669 | 1.420589872 | 0.797646886 | 0.585201999 | 0.779115393 | 0.722803671 | 0.631211307 |
| Sc006_0631.1 | 23.38675542 | 9.074995752 | 8.681565581 | 7.852252515 | 5.644478269 | 4.756624301 | 15.28393888 |
| Sc006_0637.1 | 6.664831578 | 5.593967231 | 5.398478502 | 6.969819396 | 5.520134966 | 1.844785336 | 7.545479925 |
| Sc006_0724.1 | 6.028137819 | 4.520986447 | 5.939084705 | 5.773560487 | 5.287363004 | 4.447250363 | 7.360860551 |
| Sc006_0730.1 | 3.454274932 | 5.510613414 | 3.257638255 | 4.971390703 | 3.749114312 | 2.78812164 | 4.93821826 |
| Sc006_0734.1 | 11.41919485 | 13.20354865 | 8.98878006 | 7.746477548 | 7.538363926 | 4.219056217 | 21.45367859 |
| Sc006_0737.1 | 7.067577894 | 10.6382895 | 3.406609801 | 3.330668306 | 2.50696435 | 1.747889208 | 13.61098504 |
| Sc006_0803.1 | 1.194493545 | 0 | 0 | 0 | 0 | 0 | 0 |
| Sc006_0811.1 | 6.760310531 | 3.963467566 | 4.091675262 | 5.297396127 | 4.627533408 | 7.009030526 | 10.35483657 |
| Sc006_0813.1 | 0.899302612 | 0.482238554 | 0.626630885 | 0.137307293 | 0.180527627 | 0 | 0 |
| Sc006_0816.1 | 1.197540723 | 1.284329211 | 0.069672131 | 0 | 0 | 0 | 0 |
| Sc006_0818.1 | 4.521129006 | 6.328941028 | 1.788410386 | 2.117255117 | 1.381528287 | 1.298467259 | 4.713900082 |
| Sc006_0822.1 | 1.645219498 | 1.764452281 | 2.750928334 | 3.235007122 | 3.582498932 | 4.9875705 | 2.115555119 |
| Sc006_1113.1 | 0 | 0 | 0.559609078 | 0.425212521 | 0.829126196 | 0.576940588 | 0 |
| Sc006_1115.1 | 0 | 0 | 0.875490692 | 0.578837045 | 2.258866036 | 1.119417328 | 0 |
| Sc006_1138.1 | 16.13905404 | 8.579088758 | 5.043450322 | 6.632299011 | 4.444405682 | 2.967492619 | 9.74483506 |
| Sc006_2310.1 | 5.425045441 | 5.363663054 | 2.431275644 | 4.379328062 | 2.745106988 | 4.124427206 | 5.885964839 |
| Sc006_2312.1 | 3.256492808 | 2.507434618 | 0.615068722 | 0.85237368 | 0.269213123 | 1.139103615 | 4.263250633 |
| Sc006_2336.1 | 8.15964378 | 9.851116309 | 9.286181962 | 12.54485472 | 9.511161731 | 6.106355683 | 16.37860608 |
| Sc006_2341.1 | 1.890541364 | 0.467896887 | 1.716471165 | 1.800252132 | 1.549137611 | 0.352693957 | 1.386005497 |
| Sc006_2352.1 | 13.18162578 | 10.2150706 | 8.651726723 | 8.637678014 | 6.924232583 | 1.469526207 | 15.71814169 |
| Sc006_2354.1 | 6.209470414 | 0.33297424 | 4.66409361 | 1.490011121 | 4.818038769 | 4.800206741 | 2.853332745 |
| Sc006_2658.1 | 19.98198645 | 14.31549682 | 13.49320061 | 15.30330057 | 10.3787639 | 9.616664711 | 22.66244137 |
| Sc006_2661.1 | 20.94522205 | 4.532926628 | 10.99932634 | 9.819190781 | 13.29759877 | 0.811503067 | 16.5445344 |
| Sc006_2667.1 | 3.996745818 | 5.195635199 | 2.505418579 | 3.155788357 | 2.908026184 | 3.084161242 | 1.855107977 |
| Sc006_3090.1 | 0.719734071 | 0.99892272 | 0.901458265 | 1.497093024 | 1.30001864 | 0.808590975 | 1.080807858 |
| Sc007_2790.1 | 0.684708231 | 1.37075043 | 8.092012482 | 6.864551824 | 8.24587366 | 5.936586249 | 5.453627813 |
| Sc007_3138.1 | 10.7770006 | 0.50054479 | 0 | 0 | 0 | 0.038587856 | 0.476587031 |
| Sc007_3288.1 | 1.381815158 | 0 | 0.016633054 | 0.027222629 | 0.022513107 | 0.130004883 | 0.194624402 |
| Sc008_2565.1 | 0 | 0 | 0 | 0 | 0 | 0 | 0 |
| Sc009_0142.1 | 13.78332503 | 4.305504995 | 3.043306924 | 3.877060464 | 2.321144755 | 3.285995707 | 10.79516311 |
| Sc009_0146.1 | 1.342341398 | 1.43962392 | 1.880167544 | 3.043566351 | 4.832995336 | 4.1856474 | 8.420128982 |
| Sc009_0171.1 | 10.83313761 | 7.497240105 | 7.747787319 | 8.713436864 | 7.592302022 | 11.19970269 | 11.818538 |
| Sc009_0187.1 | 9.827161873 | 11.70476839 | 11.53875822 | 13.55931515 | 12.95411824 | 10.61324483 | 12.20592341 |
| Sc009_0188.1 | 4.780781452 | 4.239845903 | 1.665757371 | 3.4335734 | 2.409505259 | 2.884702938 | 5.726782994 |
| Sc009_0283.1 | 2.29322166 | 0.723357832 | 0.991650764 | 1.825229244 | 1.277676181 | 0.736096612 | 1.515218078 |
| Sc009_0289.1 | 3.994460657 | 2.095409479 | 1.686379959 | 1.486534472 | 1.295258667 | 9.121552354 | 1.418749245 |
| Sc009_0316.1 | 0.632322149 | 1.537135392 | 0.583623749 | 0.904436873 | 0.910105514 | 2.491993738 | 0.387413713 |
| Sc009_0321.1 | 3.953830448 | 4.390033736 | 1.699041613 | 2.223062402 | 1.893038876 | 1.269132089 | 5.129891927 |
| Sc009_0323.1 | 1.233829836 | 0.155676268 | 0.746454369 | 1.176947979 | 0.726877013 | 1.386155957 | 1.037575544 |
| Sc009_0336.1 | 0 | 0.756915669 | 0.038596559 | 0.01643942 | 0.020239566 | 0 | 0.102955305 |
| Sc009_0337.1 | 0.158379205 | 4.303051716 | 2.691535398 | 6.31697805 | 3.634916035 | 7.490105874 | 4.420547896 |
| Sc009_0342.1 | 0.526702937 | 0.975691959 | 0.833565192 | 0.847886883 | 0.979482941 | 2.743469012 | 2.200244344 |
| Sc009_0344.1 | 8.284164058 | 4.392909561 | 5.341067676 | 5.819946777 | 5.22667632 | 5.692480465 | 10.76209974 |
| Sc009_0350.1 | 5.969078558 | 5.131918235 | 3.498144277 | 3.571680513 | 2.258215746 | 1.256222045 | 28.96510542 |
| Sc009_0353.1 | 3.835261138 | 0.41132112 | 1.373866388 | 1.29064255 | 1.214001083 | 6.627275051 | 4.307972968 |
| Sc009_0360.1 | 1.772125192 | 4.561332283 | 2.800226864 | 4.12393358 | 4.473765015 | 4.633601737 | 4.252532154 |
| Sc009_0363.1 | 22.37386421 | 13.754035 | 12.20367463 | 14.88983258 | 13.84298778 | 13.13197513 | 36.03678502 |
| Sc009_0365.1 | 3.968661525 | 3.496229519 | 1.787124142 | 0.416644615 | 0.88377038 | 0.257811615 | 0.434202809 |
| Sc009_0386.1 | 0.558852337 | 0.599353632 | 1.053058197 | 0.920663661 | 0.325611088 | 2.033028737 | 7.60888732 |
| Sc009_0987.1 | 1.270892625 | 1.44818441 | 0.765368365 | 0.975409795 | 1.023502442 | 0.686276198 | 1.703309293 |
| Sc009_1001.1 | 29.68170281 | 18.58135968 | 19.38065182 | 18.51590706 | 15.5700983 | 18.16219729 | 27.60882103 |
| Sc009_1107.1 | 5.891928319 | 2.241015769 | 0.695405328 | 0.832414243 | 0.911892146 | 0.965777662 | 4.232526856 |
| Sc009_1432.1 | 1.570169984 | 1.87641676 | 3.12053766 | 2.144517139 | 3.845656895 | 3.100835115 | 5.176573856 |
| Sc009_1464.1 | 4.34662929 | 0.769396787 | 0.291418367 | 0.232130379 | 0.178415434 | 1.189771402 | 0.732570867 |
| Sc009_2492.1 | 3.703074953 | 4.276940965 | 2.334021588 | 1.376192077 | 0.819722346 | 0.993075324 | 1.64828445 |
| Sc009_2878.1 | 0.969065982 | 0.929896769 | 1.000985272 | 0.937848771 | 0.542144309 | 1.113262151 | 1.666614172 |
| Sc009_2970.1 | 5.244614242 | 1.982477398 | 1.153545452 | 2.527775313 | 0.870225815 | 4.613411366 | 4.828716953 |
| Sc010_0177.1 | 0.172143734 | 0 | 0.043932424 | 0 | 0 | 2.818059636 | 0.439457188 |
| Sc010_0931.1 | 2.016477506 | 2.595139437 | 0.948815434 | 1.843280697 | 0.8954803 | 0 | 1.235463663 |
| Sc010_1495.1 | 7.705388287 | 7.050247956 | 10.49004194 | 7.369436253 | 8.567929328 | 7.203810505 | 9.023763061 |
| Sc010_1684.1 | 3.86367048 | 9.323278718 | 12.74845554 | 9.24804876 | 7.932188245 | 8.345457471 | 13.23335804 |
| Sc010_1788.1 | 7.000360856 | 3.128205359 | 0.833020694 | 0.537805494 | 0.641896381 | 1.18593343 | 4.695484412 |
| Sc010_1898.1 | 7.697310301 | 9.362986038 | 4.179527686 | 5.605156511 | 4.457207194 | 1.909211399 | 2.892219733 |
| Sc010_1905.1 | 9.729659397 | 6.122893218 | 4.226591636 | 5.516396257 | 5.553655773 | 5.338528699 | 5.70666549 |
| Sc010_1989.1 | 1.813271961 | 0.86430395 | 0.149430516 | 0.238360468 | 0.072175306 | 0.18323435 | 2.503095335 |
| Sc010_2034.1 | 12.2706283 | 6.881787025 | 8.394407211 | 8.293962266 | 7.845390699 | 3.992926945 | 12.47255378 |
| Sc010_2064.1 | 0.067857179 | 0.254712298 | 0.063501716 | 0.086559547 | 0.029652354 | 0.030856898 | 6.859877163 |
| Sc010_2086.1 | 0.443378925 | 2.340980184 | 2.553606525 | 2.648487237 | 2.519021323 | 0.899531024 | 2.820993315 |
| Sc010_2683.1 | 0.774646804 | 0.484625874 | 0.456605136 | 0.633400776 | 0.576345668 | 0.058709576 | 0.527348626 |
| Sc010_2699.1 | 7.324672531 | 4.579444207 | 5.971519389 | 7.180668142 | 6.643448566 | 1.463746551 | 7.345355328 |
| Sc010_2778.1 | 1.947977792 | 1.316452043 | 0.580221299 | 0.939671015 | 0.508628008 | 0.703794055 | 1.525929653 |
| Sc010_2784.1 | 0 | 0.176875018 | 3.28262984 | 2.80195866 | 3.82675775 | 0.419976426 | 0.94309143 |
| Sc010_2796.1 | 8.322615772 | 10.08215024 | 13.26279019 | 14.24881463 | 7.32883905 | 2.206387777 | 13.65962394 |
| Sc010_2798.1 | 10.58409997 | 9.162532533 | 6.757624431 | 7.192718013 | 3.685500159 | 2.422198252 | 13.06831443 |
| Sc010_3133.1 | 0.452250446 | 0.202094192 | 1.369869112 | 1.5150074 | 1.736739301 | 1.233919176 | 0.384842567 |
| Sc010_3142.1 | 3.217230976 | 0.800090665 | 0.682535516 | 1.681012663 | 0.675170593 | 0.636078717 | 6.380038414 |
| Sc010_3188.1 | 19.59482256 | 8.481014719 | 10.35617617 | 9.273176503 | 7.041001651 | 4.455206088 | 16.47174377 |
| Sc011_1925.1 | 7.884583363 | 2.601845224 | 3.982474501 | 5.320255742 | 4.174020085 | 7.170760275 | 6.149042659 |
| Sc011_2292.1 | 20.79535851 | 9.568024345 | 8.652440003 | 9.167357644 | 8.229598609 | 3.735836236 | 12.12490863 |
| Sc011_2455.1 | 0 | 0 | 0 | 0 | 0 | 0 | 0 |
| Sc011_2476.1 | 1.958703602 | 0.875273037 | 0.829312538 | 0.517212477 | 0.281443155 | 0.247413094 | 1.000055427 |
| Sc011_2629.1 | 10.78839644 | 0.603665529 | 0.202560712 | 0.587376794 | 0.485270092 | 1.450422181 | 2.394883599 |
| Sc011_2630.1 | 39.35391309 | 22.05764923 | 10.29560407 | 12.55639143 | 7.593537637 | 11.33383087 | 42.72139638 |
| Sc011_2633.1 | 8.716652709 | 1.44515838 | 2.273730175 | 0.671025573 | 0.529146228 | 0.229782581 | 26.7457713 |
| Sc011_2645.1 | 0 | 0 | 0 | 0 | 0 | 0 | 0 |
| Sc011_2646.1 | 16.00558335 | 10.37802244 | 6.475992619 | 5.024603184 | 2.690821194 | 3.670415305 | 93.28692097 |
| Sc011_2690.1 | 0 | 0 | 0.059372772 | 0.055465173 | 0 | 0 | 0 |
| Sc011_2692.1 | 0.044759341 | 1.392091388 | 3.048360537 | 2.852041303 | 1.249584413 | 0.936263234 | 0.685583383 |
| Sc011_2700.1 | 1.586496496 | 3.940254399 | 3.341787778 | 3.673299974 | 2.385407536 | 1.594745061 | 4.98800324 |
