# Supplementary material for: Evolution of NLR Resistance Genes in Magnoliids: Dramatic Expansions of CNLs and Multiple Losses of TNLs
Source: Front Plant Sci. 2021 Dec 21;12:777157. doi: 10.3389/fpls.2021.777157 (PMC8724549; doi:10.3389/fpls.2021.777157)
Supplement: Supplementary file 2 [file Data_Sheet_1.pdf]

Supporting information

Figure S1 The identified integrated domains (IDs) are also listed as a pie chart.

Figure S2 Chromosomal distribution of *NLR* genes in *C. kanehirae*, *C. salicifolius, P. nigrum* and *S. chinensis*.

Figure S3 A detailed ML phylogenetic tree with all sequence names. The tree was reconstructed based on the NBS domain sequences of the *NLR* genes from the seven magnoliids.

Figure S4 Reconciled *NLR* gene tree with real species phylogeny and various loss and duplication events restored.

Figure S5 (A) Conserved motifs in the N-terminal domain the four CNL subclasses. **(B)** Conserved motifs in the NBS domain of the four CNL subclasses. Larger letters indicate higher frequency.

Supporting information：

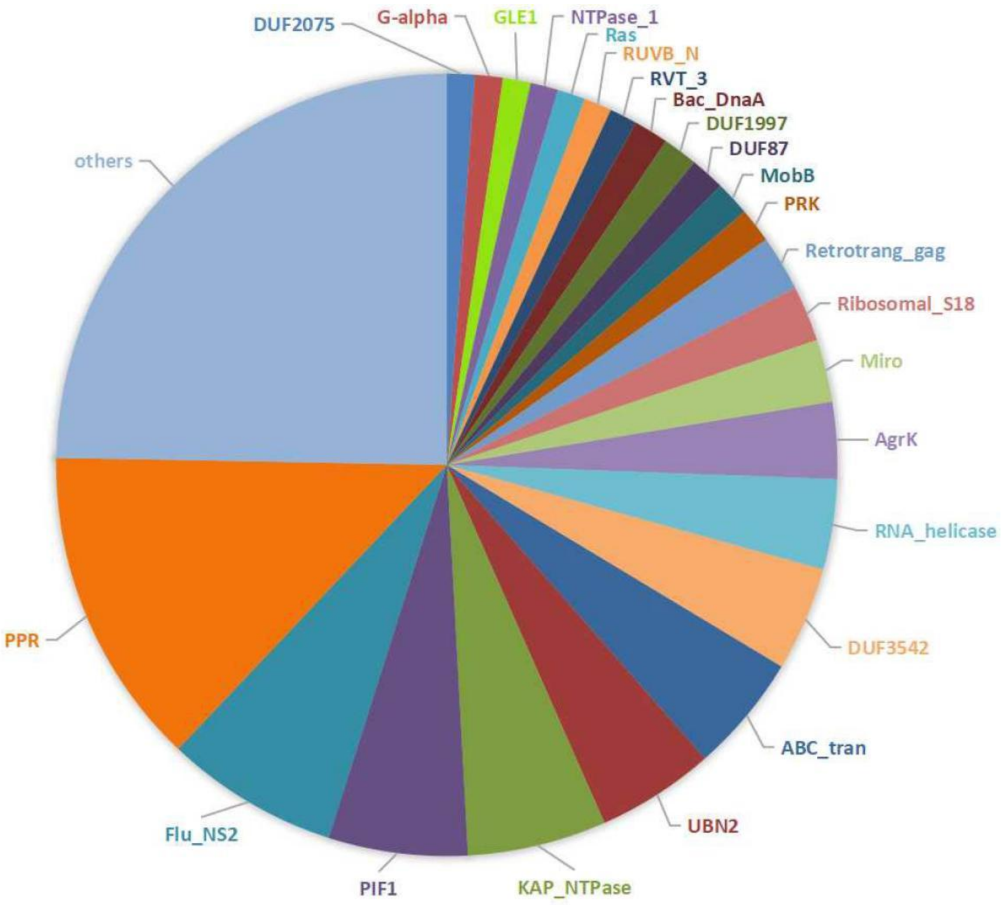

Figure S1 The identified integrated domains (IDs) are also listed as a pie chart.

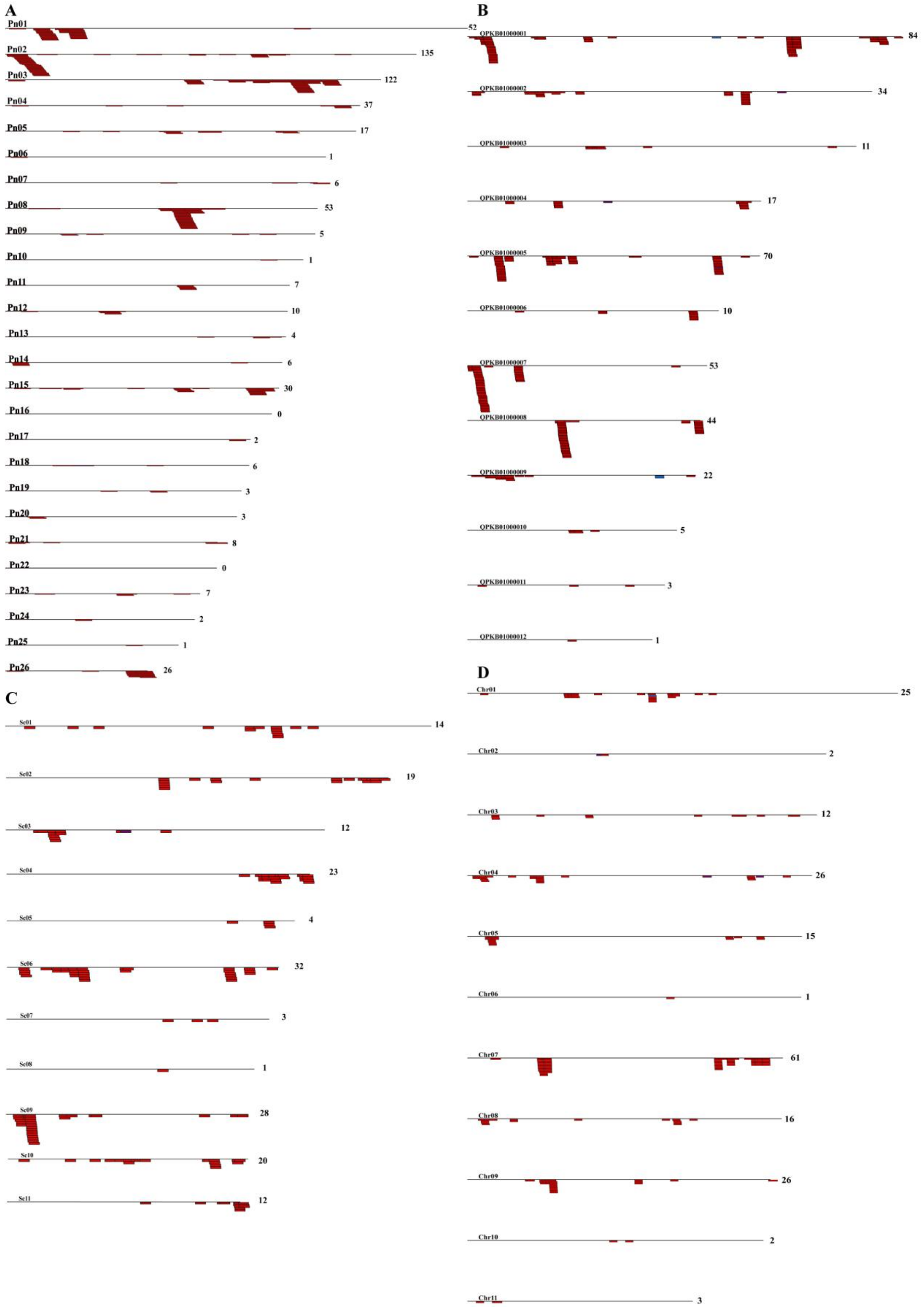

Figure S2 Chromosomal distribution of *NLR* genes in *C. kanehirae*, *C. salicifolius, P. nigrum* and *S. chinensis*.

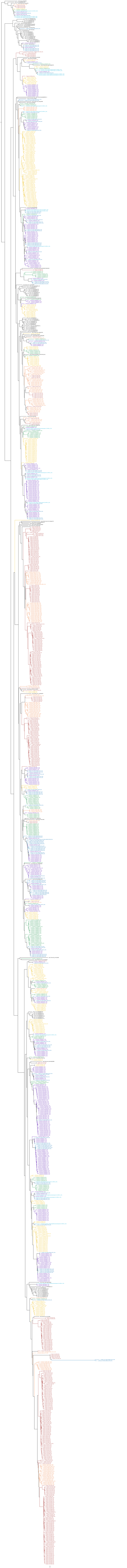

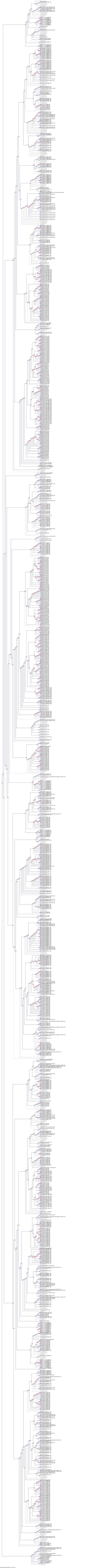

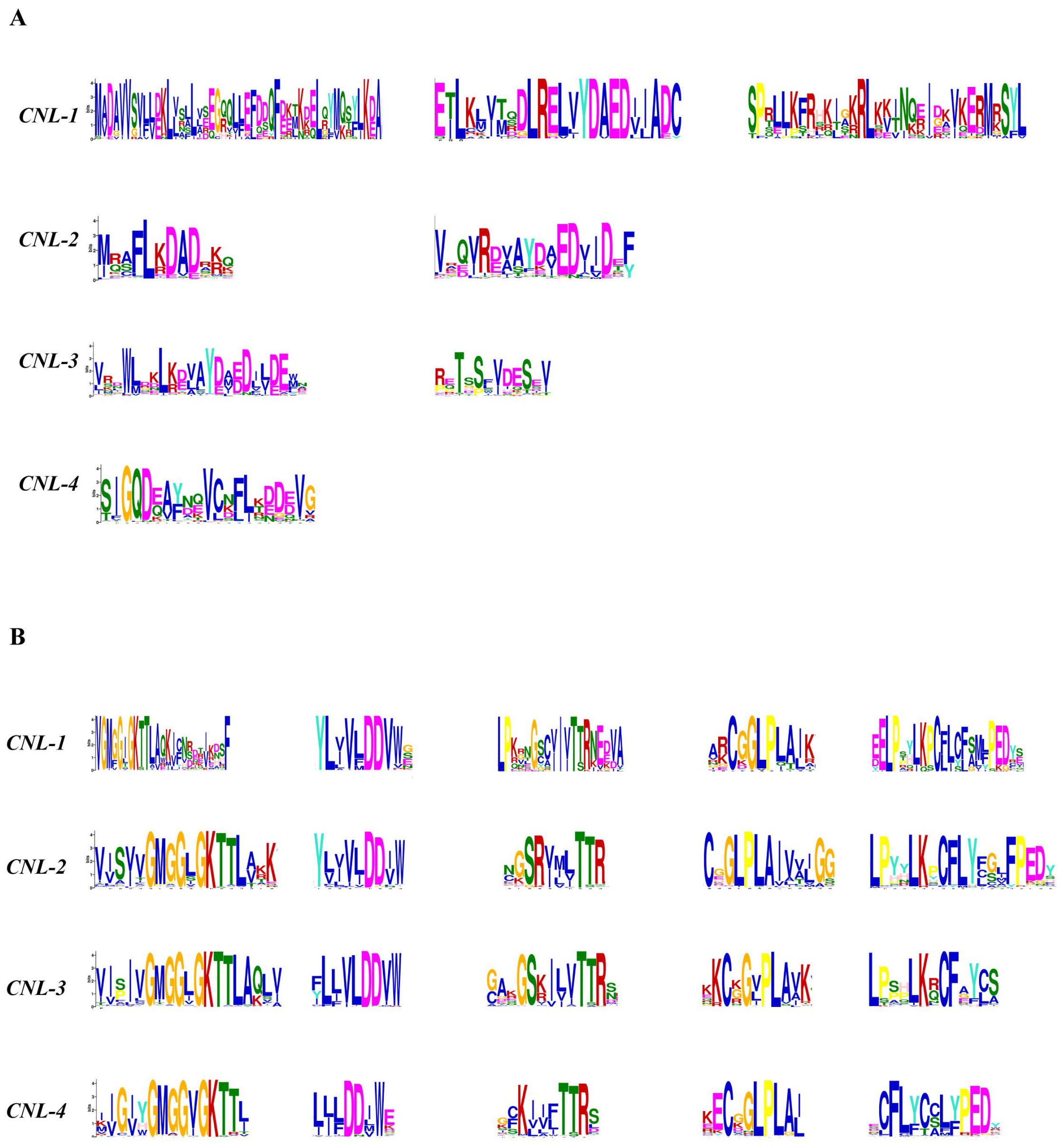

Figure S5 (A) Conserved motifs in the N-terminal domain the four CNL subclasses. **(B)** Conserved motifs in the NBS domain of the four CNL subclasses. Larger letters indicate higher frequency.
